# Structural and Functional Analysis of the CspB Protease Required for *Clostridium* Spore Germination

**DOI:** 10.1371/journal.ppat.1003165

**Published:** 2013-02-07

**Authors:** Chloe M. Adams, Brian E. Eckenroth, Emily E. Putnam, Sylvie Doublié, Aimee Shen

**Affiliations:** 1 Graduate Program in Cell, Molecular and Biomedical Sciences, University of Vermont, Burlington, Vermont, United States of America; 2 Department of Microbiology and Molecular Genetics, University of Vermont, Burlington, Vermont, United States of America; The University of Texas-Houston Medical School, United States of America

## Abstract

Spores are the major transmissive form of the nosocomial pathogen *Clostridium difficile*, a leading cause of healthcare-associated diarrhea worldwide. Successful transmission of *C. difficile* requires that its hardy, resistant spores germinate into vegetative cells in the gastrointestinal tract. A critical step during this process is the degradation of the spore cortex, a thick layer of peptidoglycan surrounding the spore core. In *Clostridium* sp., cortex degradation depends on the proteolytic activation of the cortex hydrolase, SleC. Previous studies have implicated Csps as being necessary for SleC cleavage during germination; however, their mechanism of action has remained poorly characterized. In this study, we demonstrate that CspB is a subtilisin-like serine protease whose activity is essential for efficient SleC cleavage and *C. difficile* spore germination. By solving the first crystal structure of a Csp family member, CspB, to 1.6 Å, we identify key structural domains within CspB. In contrast with all previously solved structures of prokaryotic subtilases, the CspB prodomain remains tightly bound to the wildtype subtilase domain and sterically occludes a catalytically competent active site. The structure, combined with biochemical and genetic analyses, reveals that Csp proteases contain a unique jellyroll domain insertion critical for stabilizing the protease *in vitro* and in *C. difficile*. Collectively, our study provides the first molecular insight into CspB activity and function. These studies may inform the development of inhibitors that can prevent clostridial spore germination and thus disease transmission.

## Author Summary

*Clostridium difficile* is the leading cause of health-care associated diarrhea worldwide. *C. difficile* infections begin when its spores transform into vegetative cells during a process called germination. In *Clostridium* sp., germination requires that the spore cortex, a thick, protective layer, be removed by the cortex hydrolase SleC. While previous studies have shown that SleC activity depends on a subtilisin-like protease, CspB, the mechanisms regulating CspB function have not been characterized. In this study, we solved the first crystal structure of the Csp family of proteases and identified its key functional regions. We determined that CspB carries a unique jellyroll domain required for stabilizing the protein both *in vitro* and in *C. difficile* and a prodomain required for proper folding of the protease. Unlike all other prokaryotic subtilisin-like proteases, the prodomain remains bound to CspB and inhibits its protease activity until the germination signal is sensed. Our study provides new insight into how germination is regulated in *C. difficile* and may inform the development of inhibitors that can prevent germination and thus *C. difficile* transmission.

## Introduction

The Gram-positive, spore-forming obligate anaerobe *Clostridium difficile* is the leading cause of nosocomial diarrhea worldwide [1]–[3]. The symptoms of *C. difficile*-associated disease (CDAD) range from mild diarrhea to pseudomembranous colitis and even death. Although CDAD is primarily a toxin-mediated disease [3], [4], the high cost and difficulty in treating *C. difficile* infections largely arises from its ability to form endospores [5], [6]. Because spores are metabolically dormant and intrinsically resistant to harsh physical insults [3], [7]–[9], they allow *C. difficile* to resist antibiotic treatment and persist in healthcare-associated settings. Thus, spores are the primary vectors for transmission [10] and the cause of recurrent infections, the latter of which occurs in ∼25% of cases and can lead to severe CDAD [6], [11].

In order to initiate an infection, *C. difficile* spores ingested from the environment must germinate into toxin-producing vegetative cells in the intestinal tract [1], [3], [12]. Similar to other spore-forming bacteria, *C. difficile* spores germinate specifically in response to small molecules known as germinants [13], [14]. For *C. difficile*, these germinants are bile salts, which are abundant in the small intestine [15]–[17]. While germinants have been identified for a number of bacterial species, the molecular events that occur upon germinant sensing remain poorly characterized [13], [14], [18]. Shortly after germinant addition, cortex hydrolases become activated and degrade the spore cortex, a thick protective layer of modified peptidoglycan. Because the cortex maintains the spore in a dehydrated, metabolically dormant state, the removal of this physical constraint is essential for germination to proceed and metabolism to resume in the spore core [13], [14], [18]. Nevertheless, despite the importance of cortex hydrolysis, little is known about the molecular mechanisms that regulate cortex hydrolase activity.

**Figure 1:**
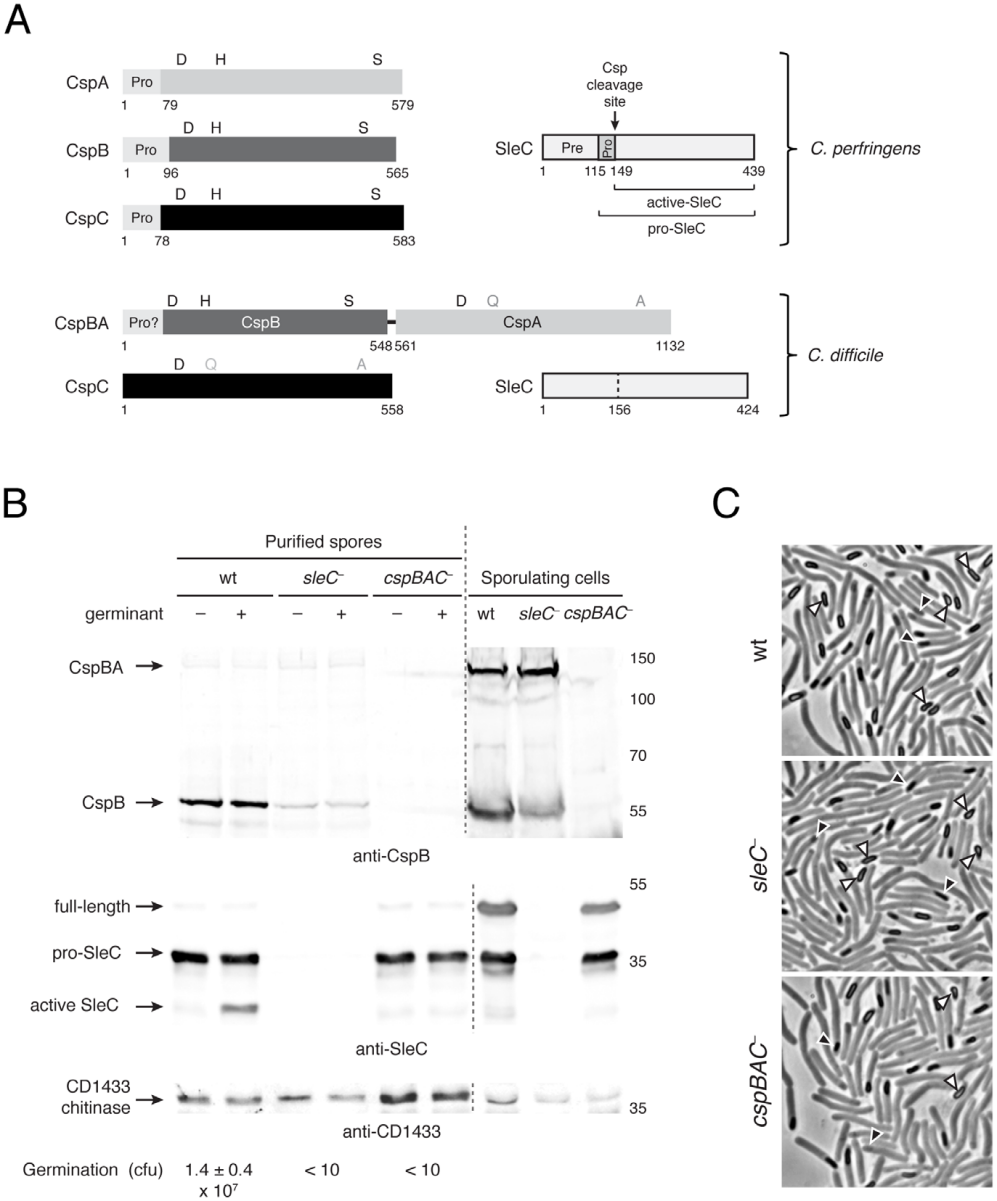
The CspBA fusion protein undergoes processing during sporulation. (**a**) Schematic of Csps and SleC in *C. perfringens* and *C. difficile*. Intact catalytic residues are black, while catalytic mutations are grey. The prodomain of *C. perfringens* Csps are shown in light grey, with their lengths indicated. The predicted prodomain of CspBA is also indicated. SleC is outlined in black, with the prepeptide (Pre), propeptide (Pro), and Csp cleavage site indicated for *C. perfringens* SleC [21], [23] (**b**) Western blot analysis of sporulating *C. difficile* and purified spores. Purified spores of the indicated strain were either untreated (−) or exposed to 0.2% w/v sodium taurocholate [16] (+, germinant) for 15 min at 37°C and analyzed by Western blotting and for germination efficiency via colony forming unit (cfu) determination. The processing products of CspB and SleC are indicated. CD1433 was previously shown to be a component of *C. difficile* spores and is used as a loading control [61]; the anti-CD1433 antiserum primarily recognizes the chitinase domain of CD1433. CspB levels were 3.5-fold lower in *sleC^−^* spores relative to wildtype spores, despite containing similar amounts of CD1433. (**c**) Phase-contrast microscopy of sporulating *C. difficile* strains used in (b) showing equivalent levels of sporulation as measured by particle counting. The white triangles indicate mature phase-bright spores that have been released from the mother cell; the black triangles highlight immature forespores in the mother cell.

In the Clostridia, the primary cortex hydrolase appears to be SleC, since disruption of *sleC* in both *C. difficile* [19] and the related foodborne pathogen *C. perfringens* [20] results in a severe germination defect. In *C. perfringens*, SleC undergoes several processing events. During sporulation, the N-terminal prepeptide is removed to produce pro-SleC, which consists of an N-terminal propeptide attached to the hydrolase domain [21]–[24]. During germination, the zymogen pro-SleC is cleaved at a conserved site to release the propeptide (Figure 1A); this event appears to activate its hydrolase activity [22], [25].

Biochemical analyses of *C. perfringens* germination exudates have shown that a fraction containing three serine proteases (CspA, CspB, and CspC) can proteolytically activate SleC hydrolase activity *in vitro* [25]. CspB alone appears sufficient to activate SleC, since the food-poisoning isolate SM101 encodes only the *cspB* gene, and disruption of this gene abrogates SleC cleavage and spore germination [26]. In the genome of *C. difficile*, three *csp* homologs are present in a bicistronic operon (*cspBA-cspC*, Figure S1), with *cspB* and *cspA* being present as a gene fusion [13]. Since disruption of the *cspBA-cspC* operon by transposon insertion results in a severe germination defect [27], cortex hydrolysis in *C. difficile* and *C. perfringens* would appear to be similarly regulated.

While studies have shown that SleC and CspB are key players during germination, the molecular mechanisms regulating their function are unknown. The sequence homology between Csp proteases (Csps) and the subtilase protease family [25] provides a starting point for understanding how Csps transduce the germination signal and activate SleC. Subtilases are serine proteases that contain a catalytic triad in the order of Asp, His and Ser [28], [29], and most subtilases are produced as pro-enzymes that autoproteolytically remove their prodomain [28], [30], [31]. While Csps purified from *C. perfringens* germination exudates are N-terminally processed [25], whether Csps are regulated in a manner analogous to other subtilases is unclear. Indeed, whether Csps actually have protease activity has not yet been directly demonstrated.

In this study, we investigated the protease activity of CspBA in *C. difficile*. By analyzing CspBA in sporulating *C. difficile* and purified spores, we demonstrate that CspBA is processed to CspB during spore assembly and that CspB undergoes autoprocessing. We also present the first crystal structure of the conserved Csp family of proteases at 1.6 Å resolution and define its key structural domains. These biochemical and mutational analyses reveal that, in contrast to previously characterized prokaryotic subtilases, wildtype CspB forms a stable complex with its prodomain. Similar to other subtilases [31], the prodomain acts as both an intramolecular chaperone and an inhibitor of CspB protease activity. These findings provide the first molecular insight into Csp function and may inform the development of strategies that can either prematurely activate *C. difficile* spore germination in the environment or prevent spore germination during disease transmission and recurrence.

## Results

### CspBA is processed during incorporation into *C. difficile* spores

The *cspBA* fusion gene is encoded in the genomes of only five clostridial species (Figure S2). *C. difficile* is unique among these species in that the CspA portion of CspBA (CD2247) lacks an intact catalytic triad (Figures 1A and S2). In order to determine whether CspBA is produced as a fusion protein, we raised antibodies against the CspB portion of CspBA and analyzed CspB production in both sporulating cells and purified spores by Western blotting. As a control, we constructed a targeted gene disruption [32] of the *cspBA-cspC* (*cd2247-cd2246*) operon (Figure S1). In sporulating *C. difficile* cells, the anti-CspB antibody detected two polypeptides of ∼130 kDa and ∼55 kDa (Figure 1B). The former corresponds to the predicted MW of CspBA of 125 kDa, while the latter corresponds to the size of Csp proteases detected in *C. perfringens* spores (∼60 kDa) [25]. Notably, the ∼55 kDa protein was enriched in purified spores, suggesting that interdomain cleavage of CspBA occurs during spore formation and that CspB may be preferentially incorporated into the developing spore. Although the mutant strains exhibited similar levels of sporulation (Figure 1C), CspB levels in *sleC^−^* mutants spores were consistently ∼3-fold lower than in wildtype spores (Figure 1B). Nevertheless, these results indicate that CspBA is processed to CspB during *C. difficile* spore assembly.

While the *cspBAC* locus was previously identified by transposon mutagenesis as being essential for *C. difficile* spore germination [27], the effect of CspBA on SleC cortex hydrolase processing was not tested. To determine whether loss of CspBA prevents SleC processing, we analyzed SleC cleavage in response to a bile salt germinant [16] by Western blotting. As predicted, disruption of *cspBAC* in *C. difficile* prevented SleC cleavage during germination (Figure 1B), and this defect could be complemented by ectopic expression of the *cspBAC* locus from a multicopy plasmid [33] (Figure S1). Thus, the *cspBAC* locus appears to regulate SleC activity in a manner similar to *C. perfringens* [20], [22], [25], [26].

### CspB undergoes autoprocessing in a position-dependent manner

**Figure 2:**
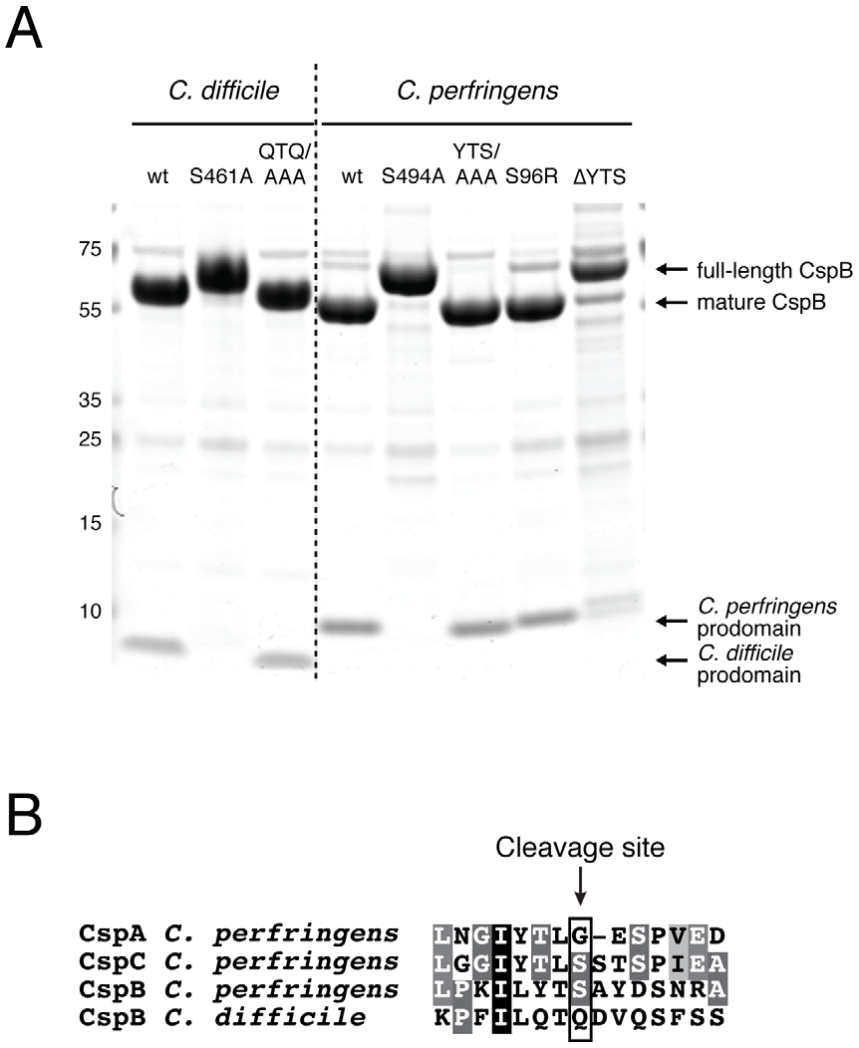
CspB undergoes autoprocessing in a position-dependent manner. (**a**) Coomassie staining of recombinant *C. perfringens* and *C. difficile* CspB variants. 7.5 µg of each purified CspB variant was resolved by SDS-PAGE on a 4–12% Bis-Tris gel and visualized by Coomassie staining. The P3-P1 residues of the prodomain were mutated to Ala for the YTS/AAA and QTQ/AAA mutants, while the P3-P1 residues were deleted from CspB *perfringens* in the ΔYTS mutant. The products resulting from autoprocessing are indicated. (**b**) Sequence alignment of Csp prodomain cleavage sites mapped by Edman sequencing; the Csp *perfringens* cleavage sites were mapped in a previous study [25]. Completely conserved identical residues are blocked in black with white text, conserved identical residues in grey with white text, and conserved similar residues in light grey.

In order to gain insight into the mechanism by which CspBA activates SleC during germination, we conducted structure-function analyses of the CspB domain of CspBA, since CspB is the only Csp protease encoded by *C. difficile* with an intact catalytic triad (Figure 1A). Based on its homology to subtilases [25] (Figure S3), we hypothesized that CspB is synthesized as a pro-enzyme that undergoes autoprocessing. To test this hypothesis, we recombinantly produced wildtype CspB *difficile* (residues 1–548 of CspBA) and CspB *perfringens*, along with mutants with the catalytic serine inactivated, and compared their apparent MW by SDS-PAGE. Whereas mutation of the catalytic serine caused both CspB *difficile* and CspB *perfringens* to run at their expected MWs of ∼60 kDa, the wildtype CspB proteins migrated with MWs of ∼55 kDa (Figure 2A). Thus, Csps autocatalytically remove their prodomain in a manner similar to other subtilases.

Using Edman degradation, we mapped the autoprocessing site of CspB *difficile* (1–548 aa) to Gln66 (data not shown). Alignment of this autocleavage site with previously mapped processing sites for CspA, CspB, and CspC of *C. perfringens* [25] revealed that Csps cleave at a similar position relative to their mature domains (Figure 2B and S4). Given the limited conservation in amino acid sequence around the Csp autoprocessing sites (Figures 2B and S4), we tested whether CspB recognizes specific amino acid residues upstream of the cleavage site. Mutation of the CspB *perfringens* P1 serine to a bulky, charged Arg did not affect autoprocessing (P1 refers to the residue N-terminal to the scissile bond based on the Schecter and Berger convention [34], Figure 2A); similarly, mutation of the P3-P1 residues to alanine did not affect CspB *perfringens* and CspB *difficile* autoprocessing (Figure 2) or the position of cleavage (data not shown). In contrast, deletion of the P3-P1 residues (ΔYTS) of CspB *perfringens* markedly reduced prodomain cleavage (Figure 2A), suggesting that the length of the prodomain affects substrate recognition or binding.

### Overall structure of CspB

**Figure 3:**
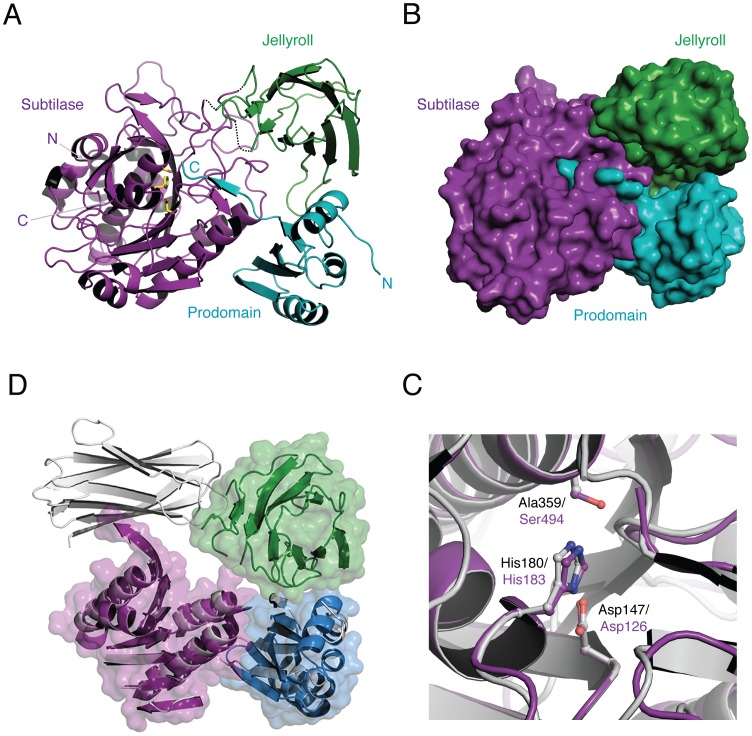
Overall structure of CspB *perfringens*. (**a**) Ribbon representation showing subtilase domain in purple, jellyroll domain in green, and prodomain in teal extending into the active site. Catalytic residues are shown as stick models with yellow carbons. (**b**) Close-up view of catalytic site. An overlay of CspB (purple) and Tk-SP (grey). The three catalytic residues are shown. Tk-SP and CspB catalytic residues are labeled in black and purple, respectively. (**c**) Space-filling model of CspB with same orientation and color scheme as (a). (**d**) Overlay of CspB (colors, same as (a)) and Tk-SP (shown in grey), showing similar overall structures with the exception of the position of the jellyroll domain. The jellyroll domains of CspB and Tk-SP are shown in green and grey, respectively. Note that only the regions with conserved secondary structure in the prodomain and subtilase domain are shown.

While these findings highlighted similarities between Csps and other subtilases, all CspB proteins capable of undergoing autoprocessing unexpectedly remained in complex with their prodomain following multiple rounds of purification (Figure 2A). In contrast, all previously characterized prokaryotic subtilases degrade their prodomain shortly after autoprocessing [31]. To gain insight into the interaction between the prodomain and subtilase domain, we determined the crystal structure of the CspB homolog from *C. perfringens*. CspB contains a subtilase domain that is similar to other subtilisin-like proteases [31], with the active site tucked within a conserved fold comprised of a six-stranded antiparallel β-sheet that is sandwiched between four conserved α-helices (Figure 3A). The catalytic triad of the active site of CspB superimposes directly with other active subtilisin-like proteases (Figure 3C), with an RMSD over the Cα atoms of only 0.11 Å between the catalytic triads of CspB and Tk-SP, the most structurally related enzyme from *Thermococcus kodakaraensis* as determined by the Dali server [35], [36].

In contrast with all previously solved prokaryotic subtilase structures, the autoprocessed prodomain stays bound to the wildtype, mature enzyme in our CspB structure. Notably, structures of prokaryotic subtilases in complex with their prodomain exist only for active site mutants [37]–[41]. The prodomain of CspB exhibits a similar structural organization to these subtilases, consisting of a 4-stranded antiparallel β-sheet and 3 α-helices (Figure 3A), with an additional β-strand extending into the catalytic cleft. The C-terminus of the CspB prodomain also extends directly into the oxyanion hole, with 19 hydrogen bonds stabilizing the intimate interaction between the C-terminal P6-P1 residues of the prodomain and the catalytic cleft (Figure S5 and Table S1 in Text S1). The prodomain-subtilase domain interface buries 1,472 Å^2^ of accessible surface area (Figure 3C).

**Figure 4:**
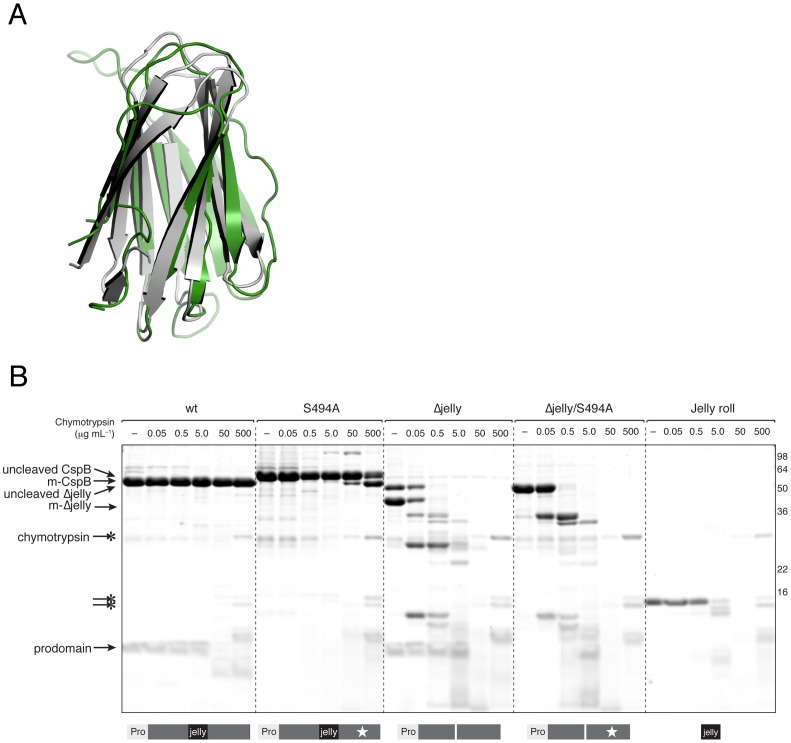
The jellyroll domain conformationally rigidifies CspB *perfringens*. (**a**) Overlay of jellyroll domain of CspB *perfringens* (green) and Tk-SP (grey). (**b**) Limited proteolysis profile of CspB and its variants. 15 µM of CspB and its variants were incubated with increasing concentrations of chymotrypsin for 60 min at 37°C. Reactions were resolved by SDS-PAGE and visualized by Coomassie staining. Schematic of CspB variants is shown below the Coomassie stained gel. “Pro” refers to the prodomain; black rectangle demarcates the jellyroll domain; thin white rectangle represents the jellyroll deletion; and white star denotes the S494A mutation. m-CspB refers to mature CspB, which is produced after autoprocessing.

A second major feature that distinguishes the structure of CspB from other subtilases is the interruption of the protease domain by an ∼130 aa insertion (Figure S3). This insertion assumes a β-barrel jellyroll fold, consisting of nine antiparallel β-strands that pack in a small hydrophobic core. The jellyroll domain interacts with both the prodomain and subtilase domain (Figure 3 and S5, Table S1 in Text S1). Although a similar jellyroll fold is present in the archaeal subtilisin Tk-SP [35] (Figures 3D and 4A, RMSD of 2.1 Å over 81 Cα atoms), the Tk-SP jellyroll domain is a C-terminal extension that interacts exclusively with the subtilase domain (Figure 3D). Nevertheless, both Tk-SP and CspB hold their jellyroll domains tightly in place with 22 and 19 bonds (primarily hydrogen bonds, Table S1 in Text S1), with a buried surface area between the jellyroll domain and subtilase domain of 1,115 Å^2^ and 1,018 Å^2^, respectively.

### The jellyroll domain rigidifies CspB

In Tk-SP, the jellyroll domain has been shown to stabilize enzyme activity at high temperatures (>90°C) [35]. To test whether the jellyroll domain might similarly stabilize CspB, we compared the susceptibility of wildtype CspB and a mutant lacking the jellyroll domain (CspB Δjelly) to limited proteolysis. *In vitro* structure-function analyses were done on CspB *perfringens* rather than *C. difficile* because the structure was solved for CspB *perfringens*. In the presence of increasing concentrations of chymotrypsin, wildtype CspB exhibited remarkable resistance to degradation even when chymotrypsin levels were approximately equimolar to CspB (0.5 mg/mL or 20 µM chymotrypsin, Figure 4B). While mutation of the catalytic serine had little effect on CspB degradation, deletion of the jellyroll domain sensitized the mutant to chymotrypsin digestion at 50 ng/mL (Figure 4B). Loss of the jellyroll domain also reduced the efficiency of CspB autoprocessing, since both uncleaved and mature CspB Δjelly were observed following purification. In contrast, only uncleaved CspB Δjelly was observed upon mutation of the catalytic serine (Figure 4B). Taken together, these results implicate the jellyroll domain in (1) positioning the prodomain to undergo autocleavage and (2) markedly restraining the conformational flexibility of CspB.

### The prodomain functions as an intramolecular chaperone

Having identified functions for the jellyroll domain, we next investigated the role of the prodomain in regulating CspB activity. For many subtilases, the prodomain acts as an intramolecular chaperone that catalyzes proper folding of the subtilase domain; once folding is complete, the mature enzyme autocatalytically separates the prodomain from its subtilase domain [30], [31]. In most subtilases, the prodomain acts as a temporary inhibitor until it is autoproteolytically removed [31], [42]. To determine the extent to which Csps follow this model of subtilase maturation, we examined the chaperone activity of the CspB prodomain. Similar to other subtilases, deletion of the prodomain dramatically reduced the solubility and yield of mature CspB, while co-expression of the prodomain *in trans* restored folding to the subtilase domain (Figure S6). The chaperone activity of the prodomain was highly specific for CspB *perfringens*, since co-expression of the prodomains of CspB *difficile* and CspC *perfringens in trans* only marginally restored folding to the subtilase domain (Figure S6).

**Figure 5:**
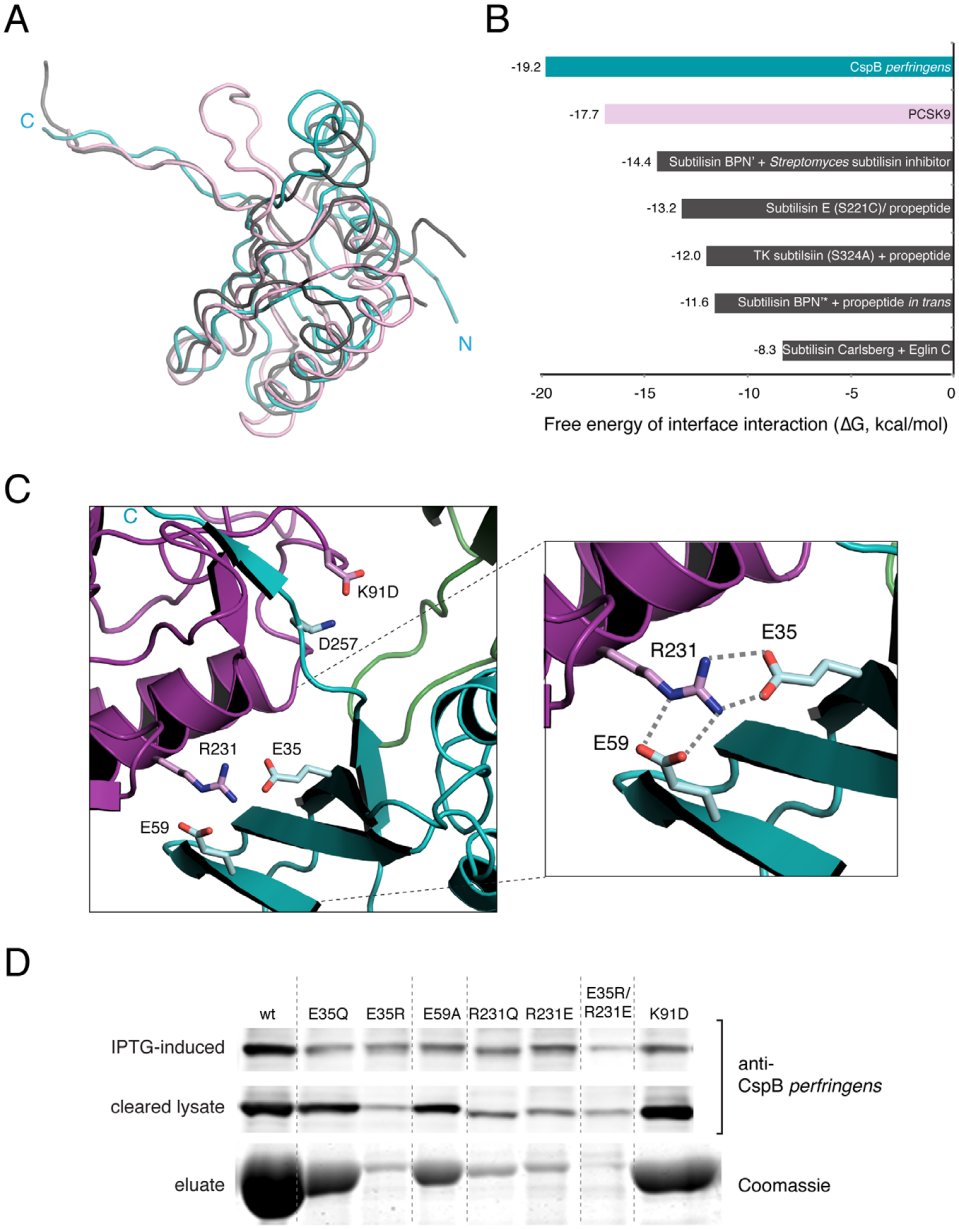
Dual salt bridges are required for prodomain intramolecular chaperone activity. (**a**) Overlay of prodomains from CspB *perfringens* (teal), Tk-SP (grey), and PCSK9 (pink). (**b**) PDBe PISA analyses of free energy of prodomain dissociation from mature subtilase, with CspB in teal, PCSK9 in pink, and others in grey. (**c**) Close-up view of dual salt-bridge interaction at prodomain-subtilase interface. The C-terminus of the prodomain (C, teal) extends toward the substrate-binding pocket. Prodomain Glu35, Glu59 and Arg91 residues are shown in teal; subtilase domain Arg231 and D257 residues are shown in magenta. (**d**) Analysis of CspB prodomain mutant solubility using Western blotting and Coomassie staining. Cultures expressing *cspB* variants were induced with IPTG, and aliquots were removed 30 minutes later (“induced-IPTG” sample). Cells were lysed by sonication and centrifuged at high speed; the “cleared lysate” sample represents the soluble fraction. CspB variants were purified by affinity chromatography. Equivalent amounts of samples were resolved by SDS-PAGE and analyzed either by Western blotting using anti-CspB *perfringens* antisera or by Coomassie staining (bottom gel, affinity-purified CspB).

Indeed, the CspB subtilase domain recognizes its prodomain with an extensive network of interactions, consisting of 27 hydrogen bonds and three salt bridges (Figure S5 and Table S1 in Text S1). The prodomain adopts a similar fold to the prodomains of related subtilisin-like proteases (Figure 5A), with the C-terminal region extending deep into the catalytic cleft (Figure 3A). The 94 Cα atoms of the prodomain align with an RMSD of 2.4 Å compared to the Tk-SP prodomain and 2.5 Å when compared to the mammalian proprotein convertase subtilisin kexin type 9 (PCSK9), respectively [43].

We compared the CspB prodomain to the PCSK9 prodomain because PCSK9 is the only other example of a wildtype subtilase that remains bound to its prodomain [43]–[45], whereas the prodomain of Tk-SP only stays bound if the catalytic serine of Tk-SP is mutated to cysteine [40], [41]. Since we did not observe any obvious structural differences to account for the difference in prodomain retention, we examined the free energy of dissociation of prodomains from their cognate subtilase domains using PDBe PISA, which is a computational server for examining interaction interfaces on proteins [46]. This analysis revealed that CspB and PCSK9 have the highest energy barriers to prodomain dissociation relative to other subtilases bound to their cognate prodomain or inhibitor (ΔG = −19.2 and −17.7 kcal/mol, respectively, Figure 5B). Interestingly, while most of the interactions holding the prodomain to the subtilase domain are not sequence specific (Table S1 in Text S1), with 15 bonds directed at backbone atoms, there are a few salt bridges that mediate specific recognition of the prodomain (Figure 5C). These salt bridges occur between Glu35/Glu59 of the prodomain and Arg231 of the subtilase domain and between Lys91 of the prodomain and Asp257 of the subtilase domain (Figure 5C). To determine the contribution of these salt bridges to CspB folding, we mutated each salt bridge residue and analyzed the effect on CspB solubility. Mutations of Glu35 to glutamine and Glu59 to alanine slightly reduced yields relative to wildtype, whereas mutation of Arg231 to glutamine strongly decreased recovery of CspB (Figure 5D), presumably because it disrupts both potential salt bridge interactions. Flipping the charges on Glu35 and Arg231 (E35R or R231E, respectively) also significantly reduced CspB yields, while swapping the Glu35-Arg231 salt bridge (E35R-R231E) failed to rescue CspB solubility. In contrast, flipping the charge on Lys91 to aspartate (K91D), which forms a salt bridge with subtilase domain residue Asp257, had little effect on K91D solubility relative to wildtype CspB (Figure 5C and 5D, Table S1 in Text S1). Taken together, these results highlight the importance of the Glu35-Arg231 and Glu59-Arg231 prodomain-subtilase domain salt bridges in promoting subtilase domain folding.

### The prodomain C-terminus sterically occludes a catalytically competent active site

**Figure 6:**
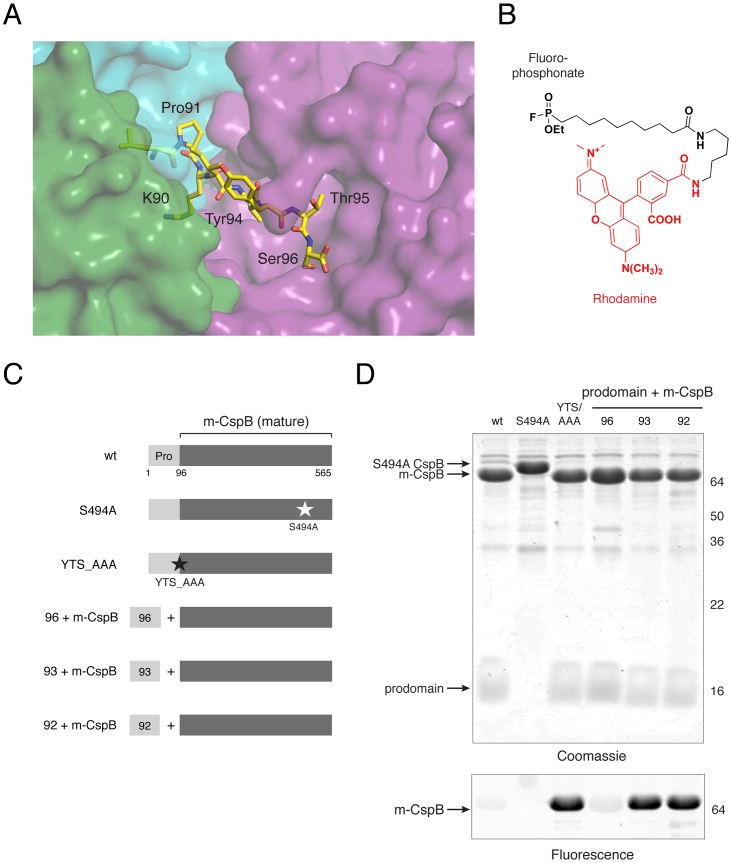
C-terminal prodomain residues sterically occlude a catalytically competent active site. (**a**) Close-up of interaction between prodomain C-terminus and substrate binding pocket. Subtilase, jellyroll and prodomains are shown in semi-transparent surface representation (purple, green, and teal, respectively). Residues 89–96 of prodomain are shown in yellow. (**b**) Structure of fluorophosphonate-rhodamine (FP-Rh) activity-based probe. Rhodamine dye is shown in red. (**c**) Schematic of CspB variants. “Pro” refers to the prodomain; “+” reflects co-expression of the prodomain in trans, with the number reflecting the prodomain length. (**d**) Labeling of CspB variants by FP-Rh. CspB variants (10 µM) were incubated with 1 µM FP-Rh probe for 20 min at RT in triplicate. The labeling reactions were resolved by SDS-PAGE on a 15% gel and visualized by fluorescent scanning followed by Coomassie staining. A single representative replicate is shown. m-CspB refers to mature CspB lacking its prodomain.

Having demonstrated the intramolecular chaperone activity of the prodomain, we next tested whether the prodomain functions as an inhibitor similar to other subtilases. Consistent with this hypothesis, the C-terminal P3-P1 residues of the prodomain bind the catalytic site in a manner analogous to an inhibitory peptide, fitting snugly within the catalytic cleft and presumably occluding access to the active site residues (Figure 6A). The S1 and S2 binding pockets perfectly accommodate the P1 serine and P2 threonine (P1 refers to the residue N-terminal to the cleavage site; S1 refers to the P1 substrate binding pocket). The bulky P3 tyrosine residue is wedged between Arg222 and Ser254 of the subtilase (Figure S5 and Table S1 in Text S1). The C-terminal P1-P3 prodomain residues form a total of 13 bonds to the S1–S3 regions of the subtilase domain. The P1 Ser96 forms seven hydrogen bonds with NE2 of catalytic His183, Ser252, Asn287, Thr493 and catalytic Ser494; P2 Thr95 forms four hydrogen bonds to different atoms of Arg222; and P3 Tyr94 forms hydrogen bonds to both the backbone amide and carbonyl of Ser254.

To test whether these residues block substrate access to the CspB active site, we used a small activity-based probe (FP-Rh, Figure 6B) to detect CspB catalytic activity. The fluorophosphonate electrophilic group of the probe reacts exclusively with catalytically competent serine hydrolases such as the subtilisins, which are a subfamily of the subtilases [47]. Nucleophilic attack by the catalytic serine results in the probe becoming covalently bound to the catalytic serine, while the rhodamine tag allows for detection of the covalently labeled enzyme by fluorescent gel scanning. Incubation of either wildtype or catalytically inactive S461A CspB with FP-Rh failed to produce detectable fluorescence, implying that the active site is inaccessible in the wildtype enzyme (Figure 6C). In contrast, mutation of the P3-P1 residues (YTS/AAA) produced a CspB variant that could be labeled on its catalytic serine, suggesting that the C-terminal prodomain residues act as gatekeepers to a catalytically competent active site. Accordingly, truncation of the C-terminal gatekeeper of the prodomain expressed *in trans* of residues YTS (P3-P1) or LYTS (P4-P1) permitted labeling of the CspB active site, whereas the full-length prodomain expressed *in trans* prevented labeling (Figure 6C). Taken together, these results indicate that the C-terminal YTS prodomain residues inhibit CspB activity.

### The CspB jellyroll domain stabilizes CspBA

**Figure 7:**
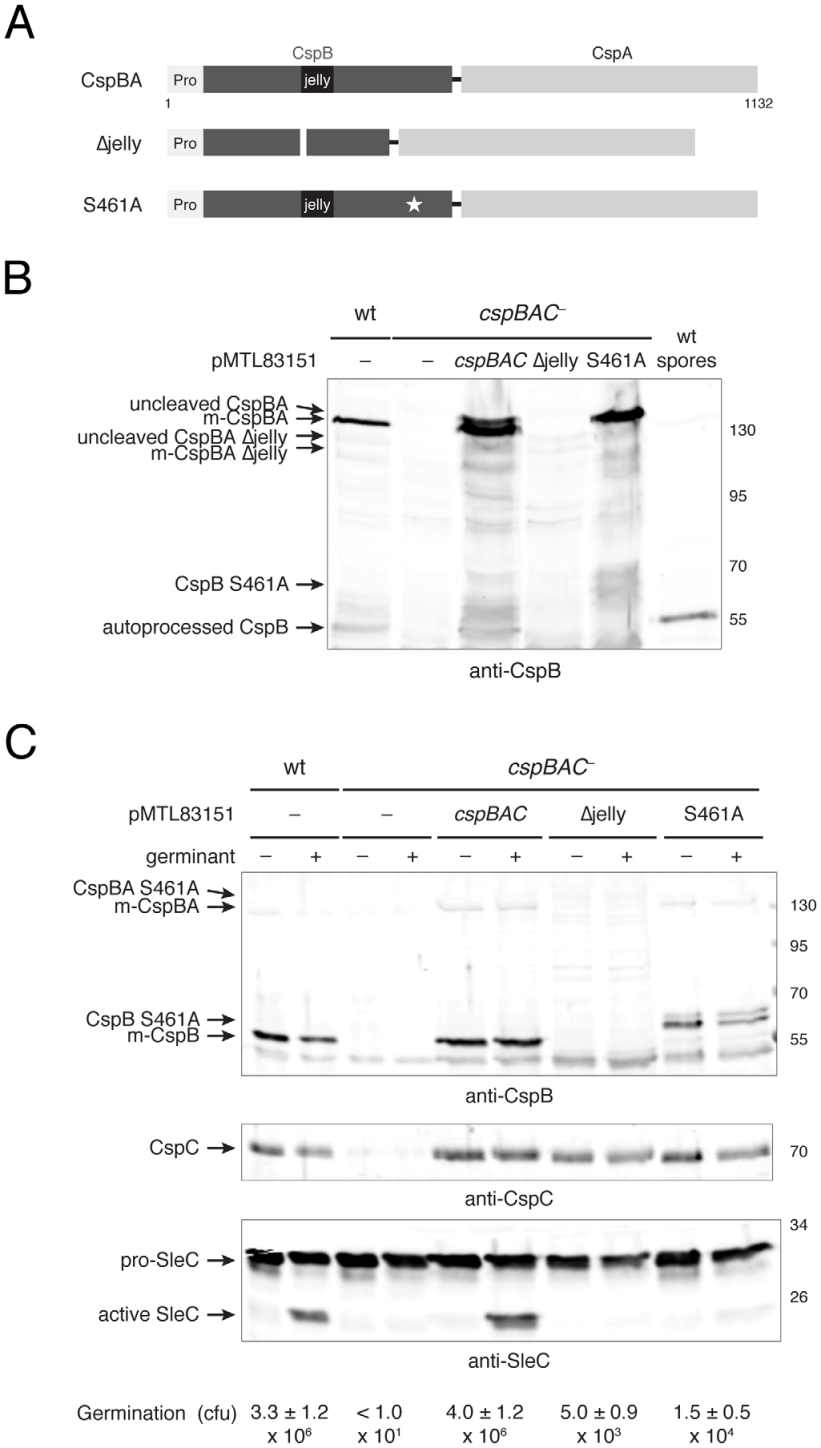
The jellyroll domain and catalytic serine of CspBA are required for efficient germination. (**a**) Schematic of CspBA variants produced by *cspBAC* complementation constructs. “Pro” denotes the prodomain; black rectangle demarcates the jellyroll domain; a thin white rectangle represents the jellyroll deletion; and white star indicates S461A mutation. (**b**) Western blot analyses of sporulating cells expressing *cspBAC* complementation constructs and (**c**) germinating spores expressing *cspBAC* complementation constructs. Purified spores of the indicated strain were either untreated (−) or exposed to 0.2% w/v sodium taurocholate (+, germinant) for 15 min at 37°C and analyzed by Western blotting with the indicted antibodies. Germination efficiency was determined via colony forming unit (cfu) determination. Representative clones of each construct are shown, but more than two clones of each complementation construct were tested. m-CspBA reflects the mature form of CspBA following autoprocessing, and m-CspB reflects the mature form of CspB following autoprocessing. The different mutant CspB variants are indicated.

Having identified key structural features of CspB *perfringens in vitro*, we next tested their functional significance in regulating CspBA activity in *C. difficile*. To this end, we cloned *cspBAC* complementation constructs in which the jellyroll domain was deleted (Δjelly, Figure 7A) or the active site serine was mutated (S461A). The *cspBAC* constructs were expressed from their native *cspBA* promoter on a multicopy plasmid (pMTL83151) [33]. Deletion of the jellyroll domain appeared to destabilize CspBA, since CspBA Δjelly levels were markedly reduced relative to wildtype and the *cspBAC* complementation strain and degradation products were apparent (Figure 7B). In contrast, mutation of the catalytic serine (S461A) did not affect CspBA levels relative to the *cspBAC* complementation strain, although CspBA S461A failed to undergo autoprocessing (Figure 7B). In purified spores, the predominant form of CspB was autoprocessed (m-CspB) in wildtype and *cspBAC-*complemented spores, whereas the predominant form of CspB in S461A spores was not autoprocessed (Figure 7C). Given that CspBA S461A was still processed at the CspB-CspA junction, an as-yet-unidentified protease apparently separates CspB from CspA.

To determine the role of CspBA autoprocessing in *C. difficile* spore germination, we examined the ability of S461A mutant spores to germinate in response to bile salts. Relative to wildtype and *cspBAC*-complemented spores, S461A mutant spores exhibited an ∼20-fold defect in germination and SleC cleavage (Figure 7C), while loss of the jellyroll domain (Δjelly) reduced spore germination by ∼70-fold (Figure 7C). Nevertheless, loss of CspBA and CspC production in the *cspBAC^−^* mutant produced a more severe phenotype than loss of the catalytic activity (S461A) or jellyroll domain (Δjelly) of CspBA. Taken together, these results indicate that CspB catalytic activity and its jellyroll domain are required for efficient *C. difficile* spore germination.

### The protease activity of CspBA is required for germination downstream of autoprocessing

The observation that ∼5% of pro-SleC undergoes cleavage during germination of S461A mutant spores (Figure 7C) raised the question as to how SleC was being activated in the absence of CspB protease activity. One possibility is that a redundant protease cleaves SleC during germination of S461A mutant spores. Another possibility is that CspB activates a second protease that directly cleaves SleC. While this latter model is more complicated, it reflects how the subtilisin-like proprotein convertase PCSK9 indirectly regulates low-density lipoprotein receptor (LDLR) levels. Rather than enzymatically degrading LDLR, PCSK9 binds and targets LDLR to the lysosome [42], [48]. However, in order to bind LDLR, PCSK9 must undergo autoprocessing to form a non-covalent complex with its prodomain; only after autoprocessing can PCSK9 recognize LDLR [42], [48]. As a result, PCSK9 is the only other wildtype subtilisin-like protease that retains its prodomain in its crystal structure following autoprocessing [43]–[45].

**Figure 8:**
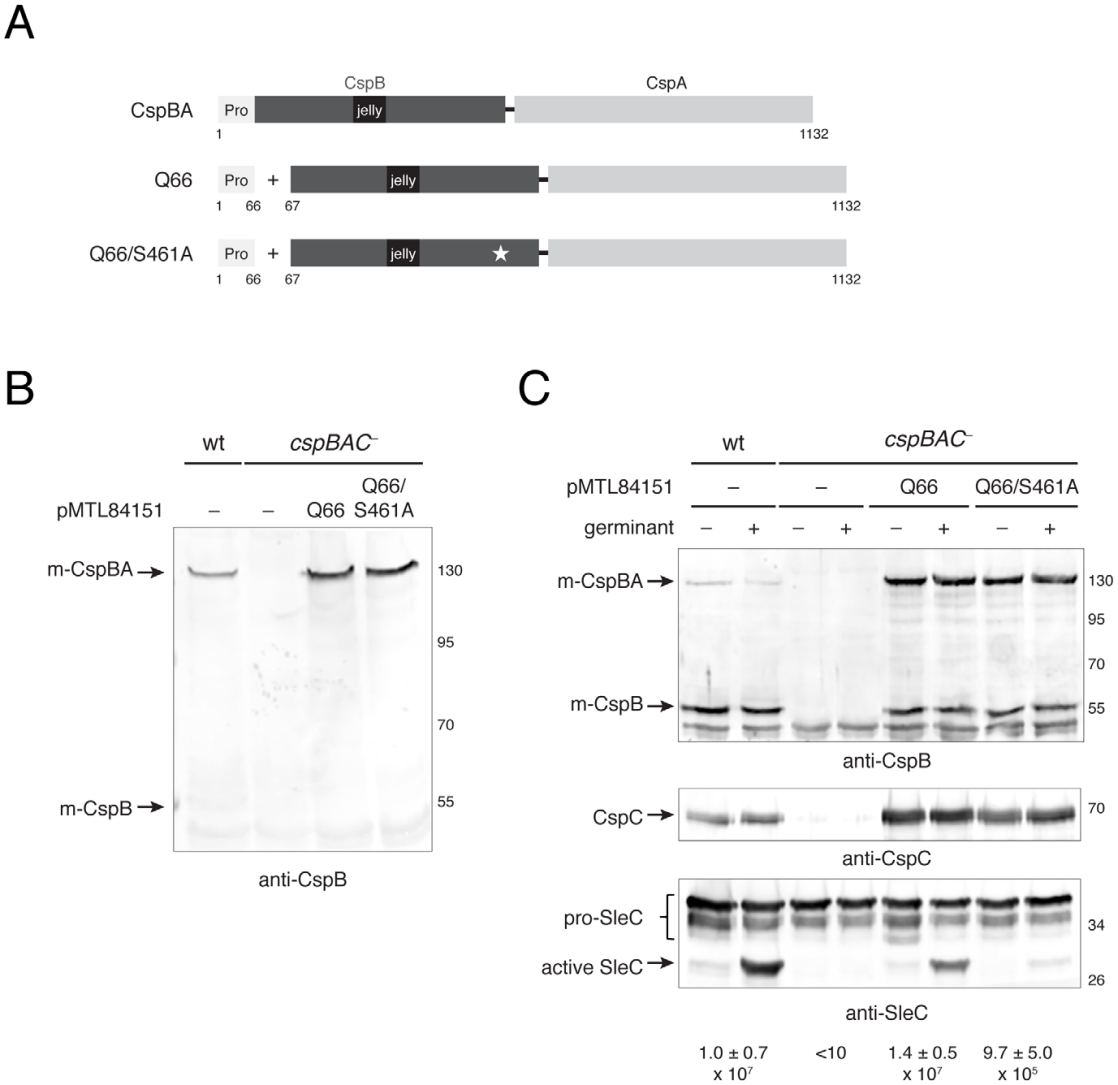
CspBA activity downstream of autoprocessing is required for efficient SleC cleavage. (**a**) Schematic of CspBA variants produced by *cspBAC* transcomplementation constructs. “Pro” denotes the prodomain; black rectangle demarcates the jellyroll domain; a thin white rectangle represents the jellyroll deletion; and white star indicates S461A mutation. (**b**) Western blot analyses of sporulating cells expressing *cspBAC* transcomplementation constructs and (**c**) germinating spores expressing transcomplementation constructs. Purified spores of the indicated strain were either untreated (−) or exposed to 0.2% w/v sodium taurocholate (+, germinant) for 15 min at 37°C and analyzed by Western blotting with the indicated antibody. Germination efficiency was determined via colony forming unit (cfu) determination. Representative clones of each construct are shown, but more than two clones of each complementation construct were tested. m-CspBA reflects the mature form of CspBA following autoprocessing, and m-CspB reflects the mature form of CspB following autoprocessing.

If CspB activity is regulated similarly to PCSK9, CspB protease activity should be dispensable once autoprocessing has occurred. To test this hypothesis, we co-expressed the CspBA prodomain with a CspBA variant lacking its prodomain such that the CspBA produced is identical to wildtype CspBA after autoprocessing (Q66, Figure 8A). The prodomain was also co-expressed with a catalytically inactive CspBA variant lacking its prodomain (Q66/S461A, Figure 8A). As predicted, Q66 and Q66/S461A transcomplementation mutants produced CspBA variants that were indistinguishable in size from wildtype in sporulating cells (Figure 8B) and purified spores (Figure 8C), although more CspBA fusion protein was observed in the transcomplementation mutant spores relative to wild type (Figure 8C). Nevertheless, Q66/S461A mutant spores exhibited a 10-fold defect in both germination and SleC cleavage relative to wildtype and Q66 mutant spores. This result indicates that the catalytic activity of CspBA is required for efficient SleC cleavage downstream of CspBA autoprocessing.

## Discussion

Spore germination is essential for *Clostridium* sp. pathogens such as *C. perfringens* and *C. difficile* to initiate infection [12], [13]. A critical step during germination is the degradation of the thick, protective cortex layer surrounding the spore core by cortex hydrolases [13], [14], [18]. However, despite their functional importance, little is known about the molecular mechanisms that control cortex hydrolase activity. In this study, we provide the first molecular insight into cortex hydrolase regulation by solving the structure of CspB, a protease required for cortex hydrolase activation. Combined with our functional analyses of CspB *in vitro* and *in vivo*, the structure reveals that Csps are subtilisin-like proteases with two distinctive functional features: a central jellyroll domain and a retained prodomain.

The central β-barrel jellyroll domain of CspB interrupts the subtilase domain and wedges itself tightly between the subtilase domain and prodomain in three-dimensional space (Figure 3C). This unique position is likely critical for CspB function, since the jellyroll domain markedly restrains the conformational mobility of CspB through extensive and specific interactions at the subtilase-jellyroll domain interface (Figure 4B and S5). The rigidity conferred by the jellyroll domain presumably helps CspB survive the environmental extremes that spores can encounter, such as freeze-thaw cycles and boiling temperatures [9]. The jellyroll domain also facilitates CspB autoprocessing *in vitro* (Figure 4B), indicative of a role in helping CspB adopt the correct subtilase fold. Consistent with this proposal, deletion of the jellyroll domain in *C. difficile* markedly reduced CspBA levels relative to wild type (Figure 7B).

In these respects, the jellyroll domain is more functionally analogous to the β-barrel P-domains of kexin-like subtilisins than to the jellyroll domain of prokaryotic Tk-SP subtilisin. Like the CspB jellyroll domain, the P-domain of kexin-like proteases, such as the mammalian enzyme furin, is important for autoprocessing, folding, stability, and activity of the subtilase domain [31], [42], [49]–[51]. In contrast, the jellyroll domain of Tk-SP is dispensable for autoprocessing, protein folding and activity *in vitro*, despite being important for Tk-SP thermostability [35].

The retention of the CspB prodomain is another unique feature identified by our study. Unlike the majority of subtilisin-like proteases, the prodomain stays bound to the wildtype subtilase domain via a network of interactions that result in tighter prodomain binding relative to other subtilases (Figure 5B and S5). Prodomain binding to its cognate protease appears highly specific, since prodomain swapping does not result in efficient folding of CspB (Figure S6). This conclusion is consistent with the limited sequence conservation of prodomains across Csps (Figure S3); indeed, even the salt bridges critical for prodomain chaperone activity (Figure 5D) are not conserved. Despite the low level of sequence conservation, the position of prodomain autoprocessing is highly conserved (Figure 2B), and a small internal deletion of the prodomain disrupts autocleavage even though diverse residues are tolerated at the P1 position (Figure 2A).

Mechanistically, Csps exhibit less specificity in P1 substrate recognition than most subtilases [52]–[56]. Nevertheless, while residues around the prodomain cleavage site do not affect autocleavage efficiency, they do control active site accessibility after autoprocessing, excluding even a small, highly reactive, serine protease probe *in vitro* (Figure 6C). Taken together, Csps appear more functionally similar to the site-specific kexin-like protease subfamily than to the highly processive subtilisin subfamily [28], [31]. Similar to kexin-like proteases, Csps cleave their putative substrate, SleC, at a single site during germination [22] (Figure 1B) and remain more closely associated with their prodomain following autoprocessing [31], [42]. By contrast, subtilisin subfamily members such as Tk-SP function as major degradative enzymes that rapidly degrade their prodomain following autoprocessing [31].

While these observations provide new insight into the structure and function of Csp proteases, they raise a number of questions for future study. Does the prodomain remain associated with autoprocessed CspB in dormant spores as it does *in vitro*? If the prodomain stays bound to mature CspB in dormant spores, what happens to the prodomain during germination? Given that chymotrypsin cannot access numerous prodomain cleavage sites during extended incubation *in vitro* (Figure 4), a significant change in CspB conformational mobility would appear to be required for the prodomain to be degraded and its putative substrate SleC to gain access to the CspB substrate binding pocket.

Another question raised by our study is the role of CspC in regulating germination in *C. difficile*. Given that Δjelly, S461A, and Q66/S461A mutant spores exhibit germination defects that are >100-fold less severe than *cspBAC^−^* spores and that a major difference between these mutant spores is the absence of CspC in *cspBAC^−^* mutant spores (Figures 7 and 8), catalytically inactive CspC (Figure 1A) may play a role in SleC activation. Recent data suggests that CspC helps transduce the germination signal to CspB (J. Sorg, personal communication). In addition, it is unclear what fraction of pro-SleC must be proteolytically activated to induce successful spore germination. Approximately 5% of spores of the CspBA catalytic mutant S461A successfully germinate, which correlates with a small fraction of pro-SleC undergoing processing in the mutant strain (Figure 8). This result suggests that only a small fraction of SleC must be proteolytically activated in order to mediate spore germination in some cells; alternatively, a small fraction of S461A spores could efficiently cleave pro-SleC and thus germinate successfully.

While further experimentation is needed, the work presented here provides the first structure-function analyses of Csp proteases *in vitro* and *in vivo* and lays the groundwork for mechanistically addressing how the germination pathway senses and integrates the germination signal. Furthermore, this study may provide the structural basis for designing therapeutics that either block prodomain and/or jellyroll domain binding to the CspBA subtilase domain during spore formation or prematurely activate CspBA to induce cortex hydrolysis. These CspBA agonists or antagonists could prevent *C. difficile* transmission and disease recurrence.

## Materials and Methods

### Bacterial growth conditions

Bacterial strains and plasmids used in this study are listed in Table S2 in Text S1. The *C. difficile* strains are isogenic with the erythromycin-sensitive strain JIR8094 [57], a derivative of the sequenced clinical isolate 630 [58]. *C. difficile* strains from freezer stocks were grown on BHIS agar plates [59] supplemented with and 0.1% sodium taurocholate (BioSynth International). To induce sporulation, *C. difficile* strains were grown on 70∶30 agar plates (63 g BactoPeptone, 3.5 g Protease Peptone, 11.1 g BHI, 1.5 g yeast extract, 1.1 g Tris base, 0.7 g NH_4_SO_4_ per liter). Media for *C. difficile* were supplemented with 10 µg thiamphenicol (Thi) mL^−1^, 50 µg kanamycin (Kan) mL^−1^, 8 µg mL^−1^ cefoxitin (TKC); 10 µg thiamphenicol mL^−1^; or 5 µg erythromycin mL^−1^ (Erm) as needed. *C. difficile* strains were maintained at 37°C in an anaerobic chamber (Coy Laboratory Products) with an atmosphere of 10% H_2_, 5% CO_2_, and 85% N_2_. *E. coli* strains were grown at 37°C in Luria-Bertrani (LB) broth. Antibiotics were used at 100 µg mL^−1^ carbenicillin for pET22b, 30 µg mL^−1^ kanamycin for pET28a, and 20 µg mL^−1^ chloramphenicol for pMTL83151 and pMTL84151 vectors in DH5α *E. coli* strains, and 100 µg mL^−1^ and 20 µg mL^−1^ in HB101 *E. coli* strains.

### Bacterial strain construction

For details, see Text S1.

### Sporulation Assay

*C. difficile* strains were inoculated from frozen stocks onto BHIS plates containing 0.1% taurocholate. After 24 hr growth, a heavy streak of the strain was transferred to a 70∶30 plate and spread uniformly across the plate. Whereas <0.1% of cells are sporulating on BHIS plates [60], ∼25% of cells undergo sporulation at the timepoints analyzed in this study as determined by phase-contrast microscopy similar to Burns *et al.* [12] (Figure 1C). While the induction of sporulation occurs at different rates within the population, this assay allows us to produce relative high rates of sporulating cells. At the indicated timepoints, cells were scraped from the plate and resuspended in PBS. The cells were pelleted and then resuspended in PBS. For Western blot analysis, 50 µL of the cell resuspension was removed, and the sample was frozen at −80°C. The remainder of the sample was analyzed by phase contrast microscopy to assess the progression of sporulation.

### Preparation of *C. difficile* samples for Western blot analysis

For sporulating *C. difficile* samples, cell pellets harvested from the sporulation assay were subject to three cycles of freeze-thaw. On the final thaw, 100 µL of EBB buffer (9 M urea, 2 M thiourea, 4% w/v SDS, 10% v/v β-mercaptoethanol) was added, and the sample was boiled with occasional vortexing for 20 min. The lysate was pelleted for 5 min at 13,000×g and then resuspended; 7 µL of 4× loading buffer (40% v/v glycerol, 0.2 M Tris pH 6.8, 20% v/v β-mercaptoethanol, 12% SDS, 0.4 mg/mL bromophenol blue) was added. The sample was boiled again for a minimum of 5 min, pelleted at 13,000×g, and 15 µL was resolved on a 7.5% (for analysis of CspB in sporulating cells) or an 11 or 12% SDS-polyacrylamide gel (for analyses of purified spores).

For analyses of purified spores, ∼5×10^6^ spores were pelleted at 15,000×g for 5 min. The spore pellet was resuspended in 50 µL EBB buffer, boiled for 20 min with periodic vortexing, pelleted at 13,000×g for 5 min, and resuspended to further solubilize proteins. Five µL of 4× loading buffer was added, and the sample was boiled for 5 min. After pelleting at 13,000×g, 10–15 µL of the sample was resolved on an 11% or 12% SDS-polyacrylamide gel.

### Western blot analysis

All antibodies used in this study were raised in rabbits by CoCalico Biologicals, with the exception of the SleC antibody, which was raised in rabbits by Pacific Immunology. The antigens used were His_6_-tagged CspB(1–548 aa), full-length His_6_-tagged CspB *perfringens*, His_6_-tagged CspC, His_6_-tagged SleC and His_6_-tagged CD1433. Samples resolved by SDS-PAGE were transferred to Immobilon-FL PVDF membranes (Millipore). The membranes were blocked in 50∶50 PBS∶LiCOR blocking buffer (LiCOR) for 30 min, after which Tween 20 was added to 0.1% v/v, and polyclonal antisera was added at 1∶1,000 for all antibodies with the exception of the anti-SleC antibody, which was used at a 1∶5,000 dilution. After a minimum of 1 hr incubation with shaking, the membranes were washed a minimum of 3 times in PBS+0.01% v/v Tween. Anti-rabbit secondary antibodies conjugated to IR800 dye (LiCOR) were added at 1∶30,000 dilution in 50∶50 PBS∶LiCOR blocking buffer containing 0.1% v/v Tween and 0.1% v/v SDS then incubated with shaking for 1 hr. The membranes were washed a minimum of 3 times in PBS+0.1% v/v Tween before imaging on an Odyssey Clx scanner (LiCOR). Western blot quantitation was performed using the indicated loading controls and LiCOR ImageStudio software.

### Purification of *C. difficile* spores

Sporulation was induced for 3–4 days on five 70∶30 plates. Spores and cell debris were scraped off the plate into 1 mL ice-cold sterile water and purified as previously described [59]. Briefly, the sample was subjected to 5 washes in ice-cold sterile water, followed by a HistoDenz gradient purification and 3–5 washes in ice-cold sterile water. Spores were stored at 4°C in water.

### Germination assay

Purified spores were enumerated using disposable semen test counting chambers (InCyto C-Chip). Approximately 5×10^7^ spores were resuspended in a total volume of 100 µL sterile H_2_O. The spores were heat activated at 60°C for 30 min, cooled for 2 min on ice, then 100 µL of 2× BHIS was added. 100 µL of the spores were removed to a tube containing 2 µL of 10% sodium taurocholate to induce germination. Both samples were incubated at 37°C for 20 min after which spores were serially diluted 10-fold into PBS. 10 µL of the dilutions was spotted onto either BHIS or BHIS+0.1% taurocholate agar plates in triplicate and incubated anaerobically at 37°C for ∼24 hr before assessing spore viability. Equivalent numbers of viable spores were recovered on untreated spores plated on BHIS+0.1% taurocholate plates and taurocholate-treated spores plated on BHIS or BHIS+0.1% taurocholate plates. Because spore clumping increased the variability in counting spores, CD1433 [61] was used as a loading control in some Western blot analyses.

### Protein Sequencing

For details see Text S1.

### Protein purification

For details see Text S1.

### Crystallization

Appropriate protein concentrations for crystallization were determined using Pre-Crystallization Test (Hampton Research, Aliso Viejo, CA). Hanging drop crystallization experiments were conducted with CspB (11 mg/mL) in 150 mM NaCl, 10 mM Tris-HCl pH 7.5 and Crystal Screen 2 (Hampton Research). Crystal trays were incubated at 12°C and initial crystal hits in 25% (v/v) ethylene glycol (Condition 3) were discovered within 24 hours. After refinement of crystallization conditions, crystals grew reproducibly to about 100*250*60 µm^3^ in 27–30% (v/v) ethylene glycol buffered to pH 5 with 50 mM sodium acetate. Crystals grew in space group P2_1_2_1_2_1_, with unit cell dimensions a = 73.87, b = 138.17, and c = 140.08 Å and two molecules in the asymmetric unit for an estimated 57% solvent content [62]–[64]. As crystallization conditions contained sufficient ethylene glycol to serve as a cryoprotectant, crystals were flash cooled in liquid nitrogen directly from the crystallization drop.

### Data collection

A complete 1.6 Å single-wavelength data set of a representative selenomethionyl-CspB crystal was collected at the selenium edge (0.9794 Å) at 100 K at the General Medical Sciences and Cancer Institutes Structural Biology Facility (GM/CA @ APS) beamline 23ID-B at the Advanced Photon Source, Argonne National Laboratory (Chicago, IL, Table S4 in Text S1).

### Data processing

Data were processed using Denzo and Scalepack [65]. Twelve selenium sites were expected, from 6 methionines in the protein sequence and two predicted molecules in the asymmetric unit, using the Matthews Coefficient program [62], [63] in the CCP4 Program Suite [64]. ShelXC/D/E, also part of the CCP4 Suite, was used to identify the selenium sites and gain initial phase information [64], [66], [67]. The 12 selenium sites and phase information were used in ShelX/E for density modification and generation of the initial phased map (Fig. S7) [66], [67].

### Structure solution and refinement

The initial model was produced by Phenix.AutoBuild using input phases from ShelX/E [66], [68]. Manual building was performed into the original phased map to reduce model bias. Refinement of the structure was done with manual building and adjustment in COOT [69] and refinement of the latest iteration of the model using Phenix.Refine [68]. All protein and ligand (non-water) B-factors were refined anisotropically. Phenix.AutoBuild with simulated annealing was used after multiple rounds of refinement to gain density for some poorly-resolved loops in the structure, resulting in the placement of several previously missing residues [68]. Ten percent of reflections were set aside for R_free_ calculation. Model was refined to an R_work_/R_free_ of 0.15/0.18 and Ramachandran statistics were 97.9% in favored regions and 2.1% in allowed regions, with no Ramachandran outliers. 957 water molecules were placed by Phenix.Refine and checked with the Check Waters feature in COOT [68], [69].

Although CspB is a dimer in the asymmetric unit, gel filtration chromatography experiments (see Text S1) indicate that CspB is a monomer in solution. In monomer 1, five residues were not built due to disorder in the electron density map (residues 411–415); in monomer 2, three residues were not built due to disorder (residues 411–413). These residues are part of a small loop located between two strands of the jellyroll domain. Additionally, the first four residues (residues 1–4) of the prodomain in each monomer were disordered and not built. Electron density for the C-terminal His_6_-tag used for protein purification (see Text S1) was seen in the second monomer only; these residues were stabilized by a crystal-packing interface, thus enabling residues 566–573 to be built in this monomer. Although the presence of calcium in the model was expected because this metal is present in many subtilisin family members [31], elemental analysis did not detect Ca^2+^ in our enzyme preparation (Dartmouth Trace Elemental Analysis Lab, data not shown). Two putative Na^+^ and three Cl^−^ atoms (confirmed by sodium iodide soaks) were placed in the model, in addition to ethylene glycol, a crystallization reagent.

### Limited chymotrypsin proteolysis

Wildtype CspB and its mutant variants were diluted to 15 µM in 10 mM Tris pH 7.5 buffer in a total volume of 150 µL. Twenty-four microliters of the mixture were transferred into 8 well strip tubes. One microliter of chymotrypsin (Sigma, 25-fold concentrate relative to indicated concentration) was added, and the mixture was mixed then incubated for 60 min at 37°C. Chymotrypsin activity was quenched by the addition of 8 µL of 4× loading buffer. The samples were boiled for 3 min at 95°C and then 7 µL was resolved on a 15% SDS-PAGE gel and visualized by Coomassie staining.

### Preparation of *E. coli* samples for Western blot analysis

*E. coli* cultures were grown as described for protein purification. One hour after IPTG induction, a 1 mL sample was removed, the OD600 measured, and the sample pelleted at 13,000×g for 2 min. Cells were lysed in 1× loading buffer (10 OD600/mL). To obtain cleared lysate samples, 30 µL of the supernatant produced upon high-speed centrifugation of sonicated lysates was added to 10 µL of 4× loading buffer. For eluate samples, 30 µL of the eluate was added to 10 µL of 4× loading buffer. All samples were boiled at 95°C for 5 min, pelleted at 13,000×g for 5 min, then 2.5 µL of induced and cleared lysate samples or 5 µL of eluate samples were resolved on a 12% SDS-PAGE gel and analyzed by Western blotting.

### Activity-based probe labeling of CspB *perfringens*

Wildtype CspB and its mutant variants were diluted to 10 µM in 10 mM Tris-HCl pH 7.5 buffer in a total volume of 155 µL. Twenty-five microliters were aliquoted in triplicate into strip tubes. 0.25 µL of 100 µM FP-Rh (fluorophosphanate-rhodamine probe) was added to CspB and incubated at RT for 10 min. Labeling was quenched by adding 8 µL 4× loading buffer to the sample and boiling at 95°C for 3 min. Six microliters of the labeling reaction was resolved on a 15% SDS-PAGE gel, and fluorescence was imaged using a Biorad PharosFX scanner.

### Accession codes

Coordinates and structure factors have been deposited in the Protein Data Bank (www.rcsb.org) under the accession number 4I0W.

## Supporting Information

Figure S1 **Csp proteases and SleC are required for spore germination in** ***Clostridium*** **sp.** (a) Schematic of *sleC* and *csp* genes in *C. perfringens* ATCC 13124 (gas gangrene isolate) [70], *C. perfringens* SM101 (food poisoning isolate) [70], and *C. difficile* 630. (b) Western blot analyses of sporulating cells and (c) germinating spores for *cspBAC^−^* complementation strains. Sodium taurocholate was used to stimulate germination for 20 min at 37°C. The number of viable spores obtained upon plating on BHIS plates containing 0.2% w/v taurocholate is given as colony forming units (cfus).
(TIF)

Figure S2 **ClustalW sequence alignment of CspBA proteins.** Completely conserved identical residues are blocked in blue, conserved identical residues in green, and conserved similar residues in yellow. A red triangle indicates catalytic triad residues (also boxed in red). Note that the catalytic His of CspB *difficile* did not align with the other CspBA homologs, despite being conserved in position in alignments with isolated CspB proteins (**Figures S3** and **S4**). Because of this discrepancy, the ClustalW alignment was altered to reflect the conservation of the catalytic His. CspBAs from *C. butyricum* (ZP_045298777), *C. botulinum* E3 (ZP_04529497), *C. acetobutylicum* (AE007820_5), *Candidatus arhtomitus* (EGX28514), and *C. difficile* 630 (YP_001088762.1). We also note that *C. tetani* E88 encodes an N-terminally truncated CspBA homolog lacking the first Asp in the catalytic triad (AAO36820), but this was not included in the alignment.
(TIF)

Figure S3 **ClustalW sequence alignment of major classes of bacterial subtilisin-like proteases.** Completely conserved identical residues (blue), conserved identical residues (green), and conserved similar residues (yellow). A red triangle indicates catalytic triad residues (boxed in red). The central insertion corresponds to the jellyroll domain. Major intracellular serine proteases (ISP): *B. subtilis* str. 168 (NP_389202.1) and *C. difficile* (YP_001088508.1); extracellular serine proteases: AprE from *B. amyloliquefaciens* (YP_003919715.1) and subtilisin E from *B. subtilis* str. 168 (NP_388911.2); CspB: *C. perfringens* ATCC 13124 (YP_697251.1) and *C. difficile* 630, 1–548 aa (YP_001088762.1).
(TIF)

Figure S4 **ClustalW sequence alignment of diverse Csp proteases.** Completely conserved identical residues are blocked in blue, conserved identical residues in green, and conserved similar residues in yellow. A red triangle indicates catalytic triad residues (also boxed in red). *C. perfringens* Csp prodomain cleavage sites are boxed in pink [25], while *C. difficile* CspB (1–548 aa) autoprocessing site is boxed in orange. α-helices and β-sheets in the CspB *perfringens* structure are indicated as a helix or a bracket above the sequence alignment, respectively. CspA *Roseburia intestinalis* (CBL08898), CspB *Blautia hansenii* (ZP_05853381), CspA *Ruminococcus gnavus* (ZP_02042115), CspA *Coprococcus catus* (CBK81001), CspC *C. perfringens* ATCC 13124 (YP_697250), CspA *C. perfringens* ATCC 13124 (YP_697252), CspB *C. perfringens* ATCC 13124 (YP_697251), and CspB *C. difficile* 630, 1–548 aa (YP_001088762.1).
(TIF)

Figure S5 **Interaction interfaces within CspB** ***perfringens*** **.** The prodomain is shown in teal, the subtilase domain in purple, and the jellyroll domain in green. Each residue involved in a predicted hydrogen bond is shown as a stick model. Bonds predicted by PDBePISA [46] are shown as dashed grey lines. All bonds predicted by PISA have been drawn, but not all are visible. (a) Prodomain interaction with mature subtilase domain, with the prodomain C-terminus extending into the active site. The Glu35/Glu59/Arg231 salt bridge interactions (**Fig. 5**) are shown, and selected residues are labeled. (b) Jellyroll domain interaction with prodomain and subtilase domain.
(TIF)

Figure S6 **Csp prodomain transcomplementation.** (a) Schematic of transcomplementation constructs. The source of the prodomain is indicated. (b) Western blot and Coomassie stain showing the purification of CspB transcomplementation mutants. Cultures expressing *cspB* variants were induced with IPTG, and aliquots were removed 30 minutes later (“induced” sample). During the purification process, a sample of the soluble fraction was removed (“cleared lysate” sample). These samples were resolved by SDS-PAGE and analyzed by Western blotting using an anti-CspB *perfringens* antibody. Following affinity purification of the His_6_-tagged CspB variants, equivalent amounts of the “eluate” were loaded and analyzed by SDS-PAGE and Coomassie staining.
(TIF)

Figure S7 **Electron density maps of CspB** ***perfringens*** **.** (a) Stereo view of bias-free, density-modified experimental map produced from SHELX/C/D/E [66] by SAD phasing using 12 selenium sites and prior to model building (map shown as dark blue mesh). The 1.6 Å map is contoured at 1 σ and shown over the C-terminal residues (92–96) of the prodomain, the catalytic triad (Asp126, His183, and Ser494), and within a 3 Å radius of each atom. Prodomain residue carbons are shown in cyan and catalytic residue carbons in yellow. OXT indicates the prodomain C-terminus resulting from proteolytic cleavage. (b) 1.6 Å resolution anomalous electron density map from SHELX/C/D/E showing selenium anomalous signal (orange mesh) in selenomethionine (MSe) residues. Map is contoured at 3 σ and shown over MSe237 and MSe558.
(TIF)

Text S1 **The file includes supplemental** **Materials and Methods** **, Tables S1, S2, S3, S4, and associated references.**
(DOC)
