# Supplementary figures and images for: Structural and Functional Analysis of the CspB Protease Required for Clostridium Spore Germination
Source: PLoS Pathog. 2013 Feb 7;9(2):e1003165. doi: 10.1371/journal.ppat.1003165 (PMC3567191; doi:10.1371/journal.ppat.1003165)

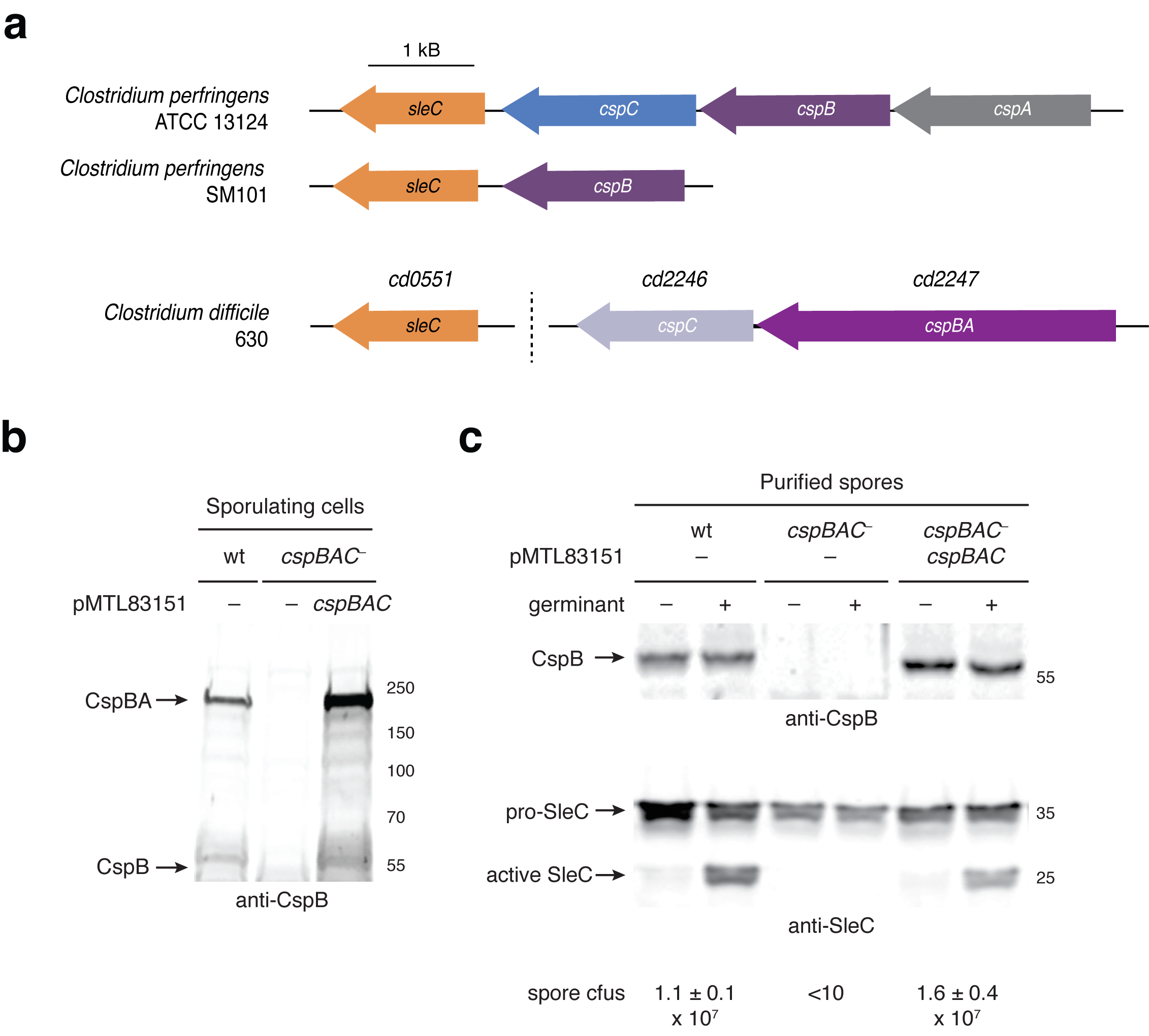

Supplement: Figure S1 — Csp proteases and SleC are required for spore germination in Clostridium sp. (a) Schematic of sleC and csp genes in C. perfringens ATCC 13124 (gas gangrene isolate) [70], C. perfringens SM101 (food poisoning isolate) [70], and C. difficile 630. (b) Western blot analyses of sporulating cells and (c) germinating spores for cspBAC− complementation strains. Sodium taurocholate was used to stimulate germination for 20 min at 37°C. The number of viable spores obtained upon plating on BHIS plates containing 0.2% w/v taurocholate is given as colony forming units (cfus). (TIF) [file ppat.1003165.s001.tif]

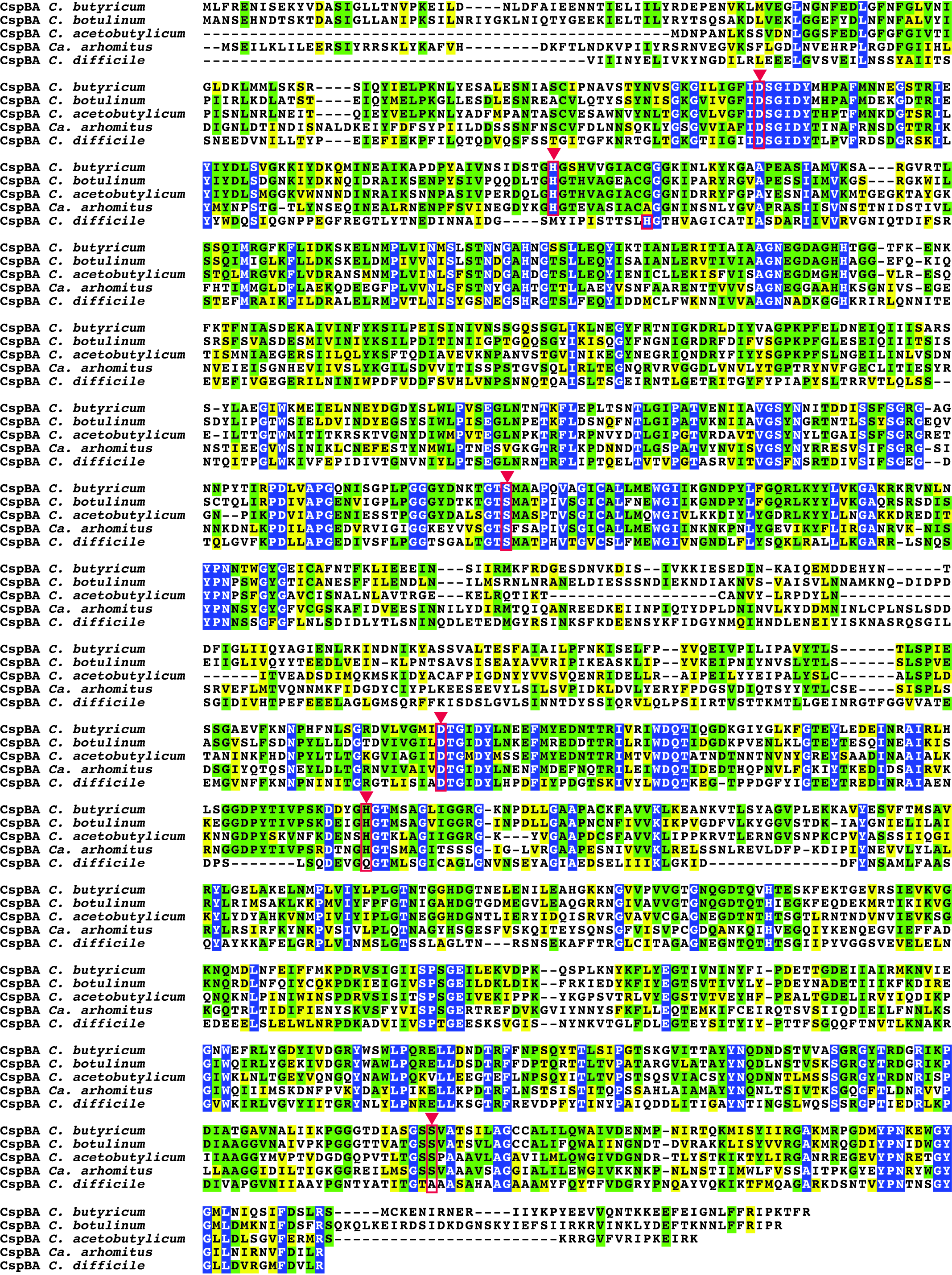

Supplement: Figure S2 — ClustalW sequence alignment of CspBA proteins. Completely conserved identical residues are blocked in blue, conserved identical residues in green, and conserved similar residues in yellow. A red triangle indicates catalytic triad residues (also boxed in red). Note that the catalytic His of CspB difficile did not align with the other CspBA homologs, despite being conserved in position in alignments with isolated CspB proteins (Figures S3 and S4). Because of this discrepancy, the ClustalW alignment was altered to reflect the conservation of the catalytic His. CspBAs from C. butyricum (ZP_045298777), C. botulinum E3 (ZP_04529497), C. acetobutylicum (AE007820_5), Candidatus arhtomitus (EGX28514), and C. difficile 630 (YP_001088762.1). We also note that C. tetani E88 encodes an N-terminally truncated CspBA homolog lacking the first Asp in the catalytic triad (AAO36820), but this was not included in the alignment. (TIF) [file ppat.1003165.s002.tif]

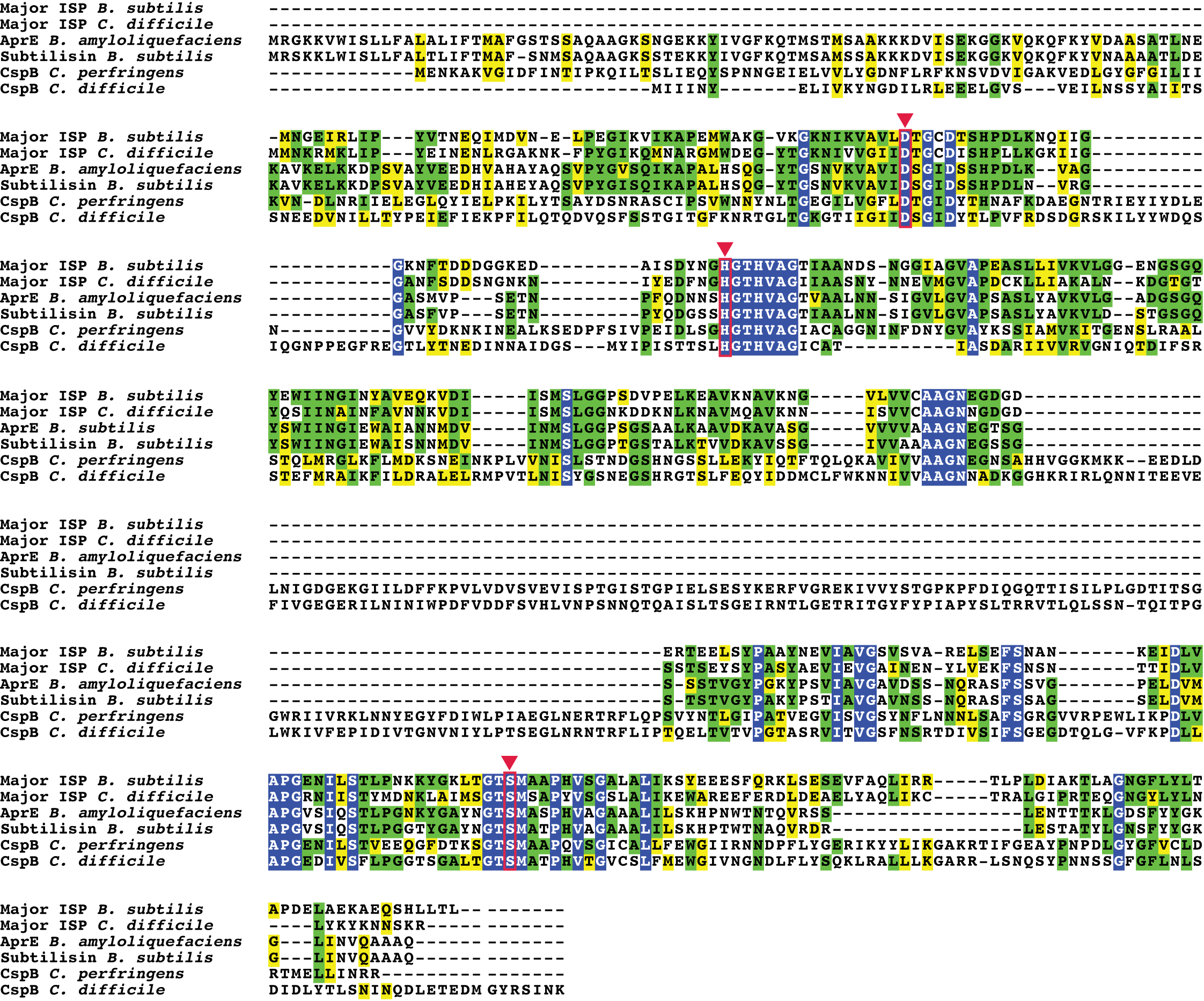

Supplement: Figure S3 — ClustalW sequence alignment of major classes of bacterial subtilisin-like proteases. Completely conserved identical residues (blue), conserved identical residues (green), and conserved similar residues (yellow). A red triangle indicates catalytic triad residues (boxed in red). The central insertion corresponds to the jellyroll domain. Major intracellular serine proteases (ISP): B. subtilis str. 168 (NP_389202.1) and C. difficile (YP_001088508.1); extracellular serine proteases: AprE from B. amyloliquefaciens (YP_003919715.1) and subtilisin E from B. subtilis str. 168 (NP_388911.2); CspB: C. perfringens ATCC 13124 (YP_697251.1) and C. difficile 630, 1–548 aa (YP_001088762.1). (TIF) [file ppat.1003165.s003.tif]

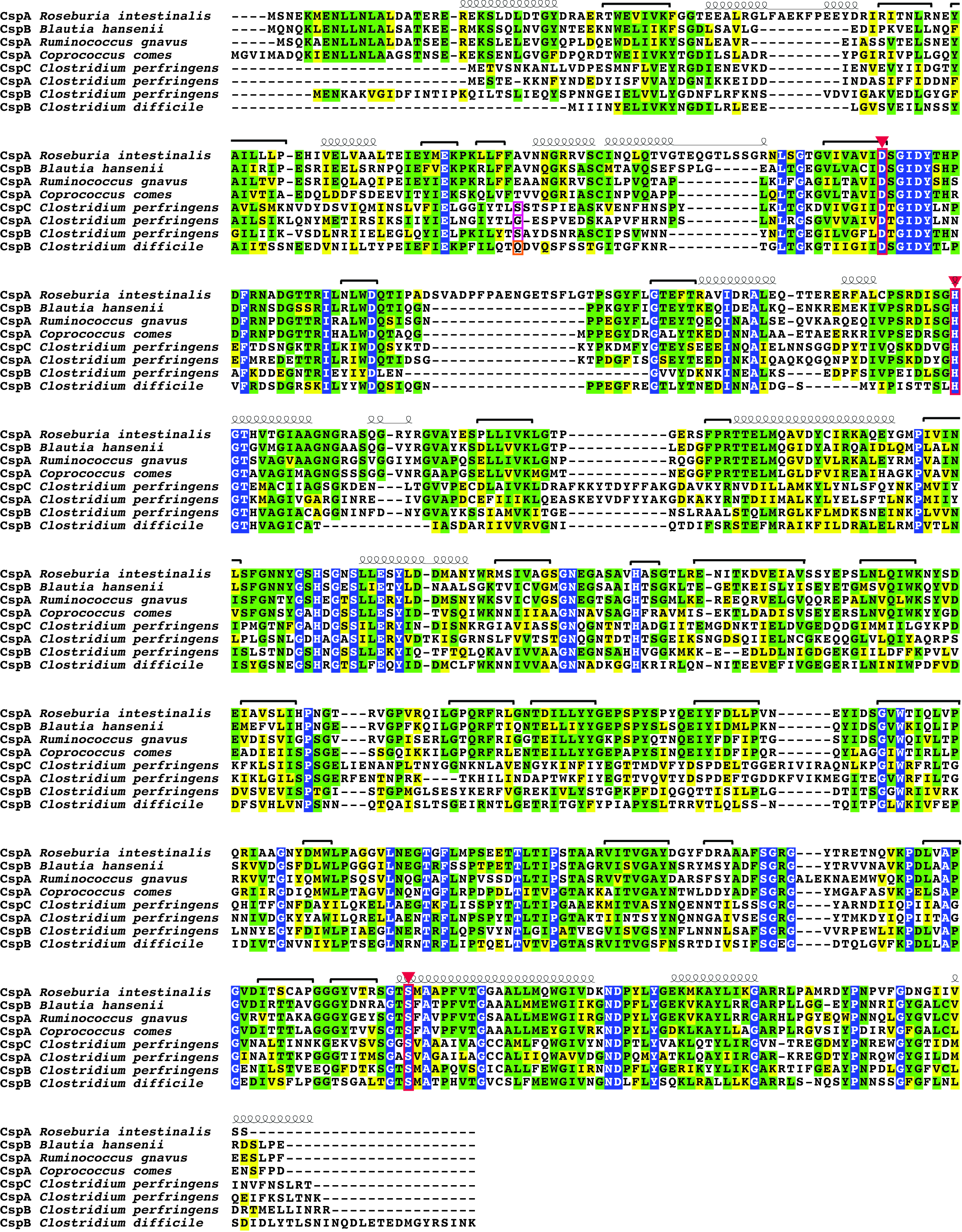

Supplement: Figure S4 — ClustalW sequence alignment of diverse Csp proteases. Completely conserved identical residues are blocked in blue, conserved identical residues in green, and conserved similar residues in yellow. A red triangle indicates catalytic triad residues (also boxed in red). C. perfringens Csp prodomain cleavage sites are boxed in pink [25], while C. difficile CspB (1–548 aa) autoprocessing site is boxed in orange. α-helices and β-sheets in the CspB perfringens structure are indicated as a helix or a bracket above the sequence alignment, respectively. CspA Roseburia intestinalis (CBL08898), CspB Blautia hansenii (ZP_05853381), CspA Ruminococcus gnavus (ZP_02042115), CspA Coprococcus catus (CBK81001), CspC C. perfringens ATCC 13124 (YP_697250), CspA C. perfringens ATCC 13124 (YP_697252), CspB C. perfringens ATCC 13124 (YP_697251), and CspB C. difficile 630, 1–548 aa (YP_001088762.1). (TIF) [file ppat.1003165.s004.tif]

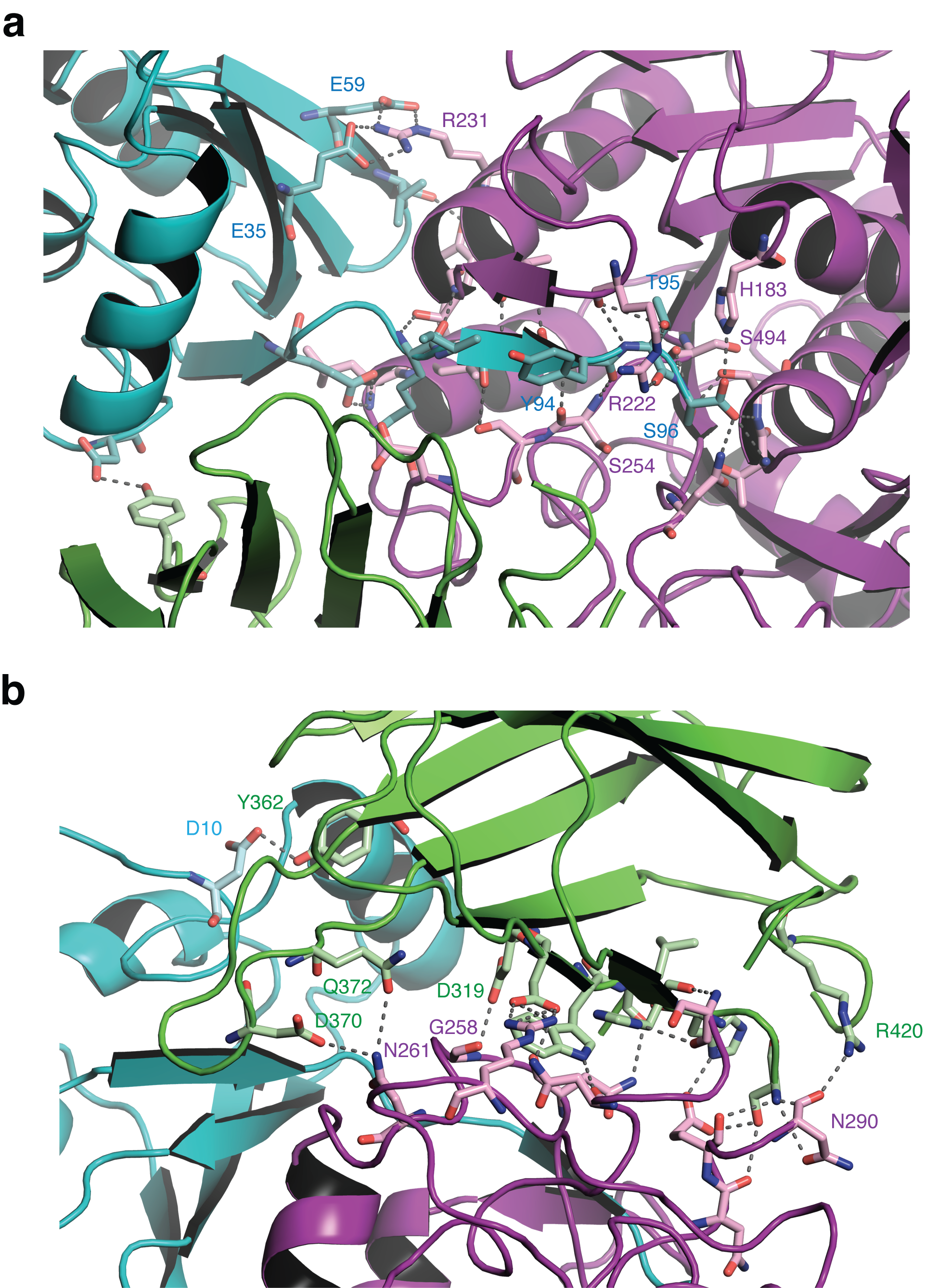

Supplement: Figure S5 — Interaction interfaces within CspB perfringens . The prodomain is shown in teal, the subtilase domain in purple, and the jellyroll domain in green. Each residue involved in a predicted hydrogen bond is shown as a stick model. Bonds predicted by PDBePISA [46] are shown as dashed grey lines. All bonds predicted by PISA have been drawn, but not all are visible. (a) Prodomain interaction with mature subtilase domain, with the prodomain C-terminus extending into the active site. The Glu35/Glu59/Arg231 salt bridge interactions ( Fig. 5 ) are shown, and selected residues are labeled. (b) Jellyroll domain interaction with prodomain and subtilase domain. (TIF) [file ppat.1003165.s005.tif]

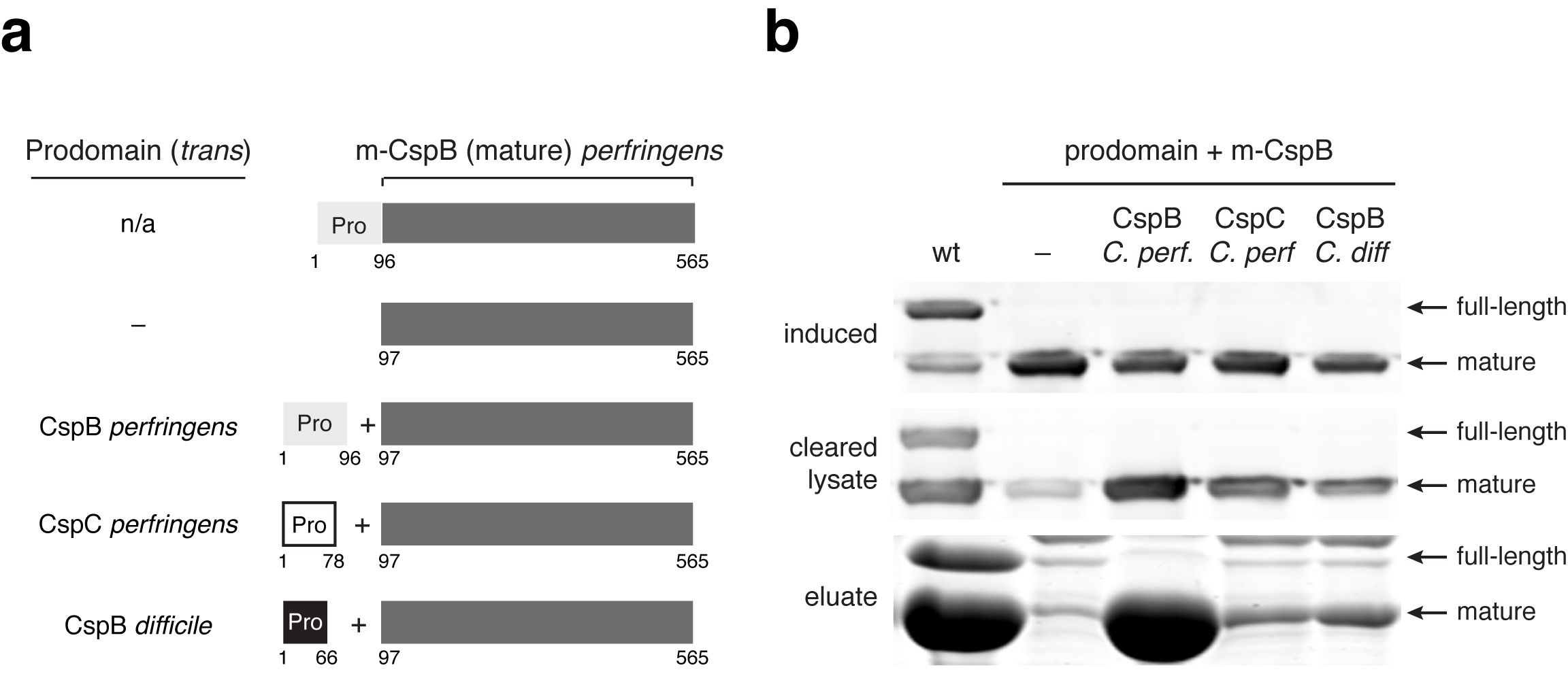

Supplement: Figure S6 — Csp prodomain transcomplementation. (a) Schematic of transcomplementation constructs. The source of the prodomain is indicated. (b) Western blot and Coomassie stain showing the purification of CspB transcomplementation mutants. Cultures expressing cspB variants were induced with IPTG, and aliquots were removed 30 minutes later (“induced” sample). During the purification process, a sample of the soluble fraction was removed (“cleared lysate” sample). These samples were resolved by SDS-PAGE and analyzed by Western blotting using an anti-CspB perfringens antibody. Following affinity purification of the His6-tagged CspB variants, equivalent amounts of the “eluate” were loaded and analyzed by SDS-PAGE and Coomassie staining. (TIF) [file ppat.1003165.s006.tif]

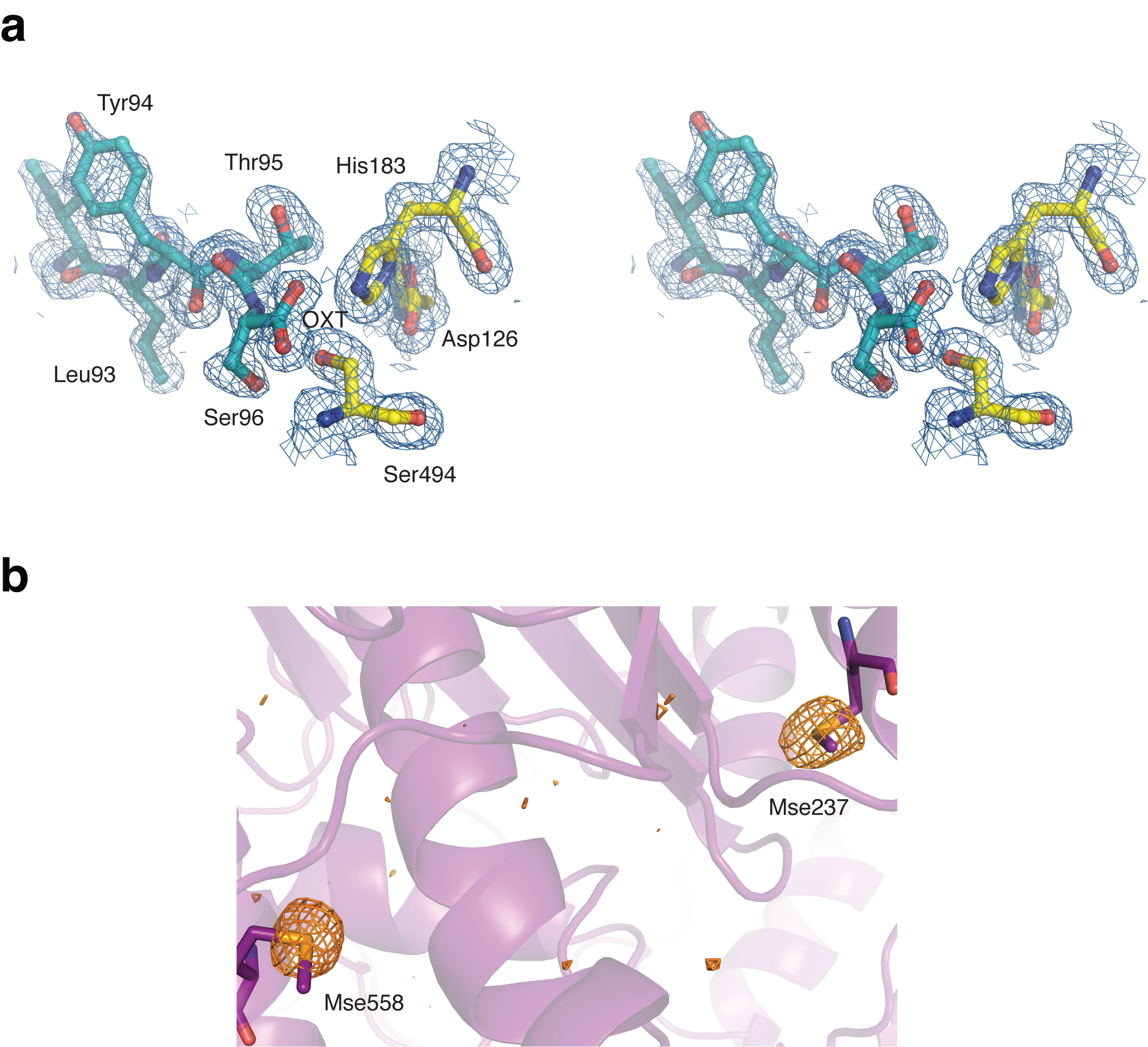

Supplement: Figure S7 — Electron density maps of CspB perfringens . (a) Stereo view of bias-free, density-modified experimental map produced from SHELX/C/D/E [66] by SAD phasing using 12 selenium sites and prior to model building (map shown as dark blue mesh). The 1.6 Å map is contoured at 1 σ and shown over the C-terminal residues (92–96) of the prodomain, the catalytic triad (Asp126, His183, and Ser494), and within a 3 Å radius of each atom. Prodomain residue carbons are shown in cyan and catalytic residue carbons in yellow. OXT indicates the prodomain C-terminus resulting from proteolytic cleavage. (b) 1.6 Å resolution anomalous electron density map from SHELX/C/D/E showing selenium anomalous signal (orange mesh) in selenomethionine (MSe) residues. Map is contoured at 3 σ and shown over MSe237 and MSe558. (TIF) [file ppat.1003165.s007.tif]
