# Supplementary material for: Structural and Functional Analysis of the CspB Protease Required for Clostridium Spore Germination
Source: PLoS Pathog. 2013 Feb 7;9(2):e1003165. doi: 10.1371/journal.ppat.1003165 (PMC3567191; doi:10.1371/journal.ppat.1003165)
Supplement: Text S1 — The file includes supplemental Materials and Methods , Tables S1, S2, S3, S4, and associated references. (DOC) [file ppat.1003165.s008.doc]

**Text S1**

**Supporting Materials and Methods**

**Strain and plasmid construction for genetic manipulation of *C. difficile***. Oligonucleotides used in this study are listed in Supporting Table 3. *C. difficile* strain 630 DNA was used as a template for PCR amplification. Sequencing was performed at the University of Vermont DNA Analysis Core Facility. To construct the null mutation in the *cspBAC* locus, the ClosTron method for targeted gene disruption in *C. difficile* was used. A modified plasmid containing the retargeting group II intron, pCE240 (a gift from C. Ellermeier, University of Iowa), was used as the template with primers #533, 534, and 535 and the EBS Universal primer (#532), as outlined in the TargeTron users’ manual (Sigma Aldrich). The resulting retargeting sequence was digested with BsrGI/HindIII and cloned into pJS107 (a gift from J. Sorg, University of Texas A&M), which is a derivative of pJIR750ai (Sigma Aldrich) with similarity to pMTL007 . The resulting plasmid pJS107-*cspBAC-*81 was transformed into HB101/pRK24 for conjugation into JIR8094 to generate *cspBAC–*. Specifically, the *E. coli* donor strain was grown aerobically in 2 mL of LB supplemented with ampicillin and chloramphenicol until early stationary phase. The culture was pelleted gently at 2,500 x g for 5 min, and the resulting pellet was transferred into the anaerobic chamber. The pellet was resuspended in 1 mL of a *C. difficile* recipient strain grown anaerobically in BHIS at 37˚C with slow shaking until early stationary phase. 100 µL of the cell mixture was spotted onto a single BHIS agar plate and incubated overnight for 14 – 18 hr after which the cells were scraped into 2.5 mL BHIS. Seven 100 µL drops of the mixture were spotted onto three BHIS/TKC plates. Thiamphenicol-resistant colonies typically appeared with 30 – 48 hr, after which they were streaked onto BHIS plates containing Thi, Kan and 50 µM FeSO­4. This step induces the ferroredoxin promoter that controls the expression of the group II intron. The resulting streaks were transferred to BHIS-Erm plates; Erm-resistant colonies typically appeared after 48 hr, after which they were struck to single colonies then screened by PCR using primers #457 and 538, the latter of which binds within the pJS107 vector.

To complement the targetron disruption of *cspBAC*, a 5364 bp fragment containing the *cspBAC* operon and its upstream region was amplified using primers #691 and 665. The resulting PCR product was digested with NotI-HF and XhoI (New England Biolabs) and ligated into pMTL83151 . The S461A mutation was introduced using PCR splicing by overlap extension (SOE) . Primer pair #691 and 464 were used to amplify the 5’ SOE product, while primer pair #665 and 465 were used to amplify the 3’ SOE product. The resulting fragments were mixed together, and the flanking #691 and #665 primers were used to amplify the 5364 bp S461A fragment. The ∆jelly mutation (corresponds to deletion of aa 260-392) was constructed similar to the S461A mutation, with the exception that the internal SOE primers were #746 (used with #691) and #745 (used with #665). All complementation plasmids were transformed into HB101/pRK24, and the conjugation was performed as described except that after 14 hr incubation on non-selective media, the bacterial mixture was scraped into 1 mL PBS. 100 µL of the mixture was spotted onto a total of five BHIS-TKC plates. Thiamphenicol-resistant colonies typically appeared after 48 hr incubation, after which they were re-streaked to single colonies. At least two independent clones of each strain constructed were analyzed.

The *sleC* mutant was constructed as previously described by Burns *et al*. using primers #641, 642, and 643, with the exception that pJS107 was used for the targeting vector. Primer pair #461 and 463 were used to screen colonies for clones containing the targetron insertion.

To construct *cspBAC* pMTL84151 complementation constructs, PCR SOE was used to introduce the following sequence, TAGGAGGGATTTATG between the codons for Gln66 and Asp70. This sequence is derived from the end of the *cspBA* gene, including the stop codon, and the start of the *cspC* gene, including the start codon. Insertion of this sequence results in the introduction of a stop codon, ribosome binding site and start codon in between the codons for Gln66 and Asp70. In particular, primers #691 and #951 were used to amplify the 5’ SOE product, while primers #864 and #950 were used to amplify the 3’ SOE product using either pMTL83151-*cspBAC* or pMTL83151-*cspBAC* S461A as the PCR template. The resulting fragments were mixed together, and the flanking #691 and #864 primers were used to amplify a 2790 bp fragment. The fragment was gel-purified, digested with NotI and KpnI and ligated to gel-purified pMTL83151-*cspBAC* digested with the same enzymes. The resulting pMTL83151-Q66 and Q66/S461A plasmids were used as PCR templates for a second PCR reaction, this time using primers #691 and #665. The resulting PCR fragment was gel purified, digested with NotI and XhoI, and ligated to pMTL84151 digested with the same enzymes. The pMTL84151 multicopy vector was used for the transcomplementation because expression of the split prodomain constructs from the pMTL83151 multicopy vector resulted in lower levels of CspBA relative to wildtype (data not shown). This observation likely reflects a reduced efficiency in prodomain intermolecular chaperone activity relative to intramolecular chaperone activity. pMTL84151 plasmids were conjugated into *C. difficile* using the same method as conjugating pJS107 plasmid constructs.

**Construction of CspB constructs** **for recombinant protein purification**. Primers used are listed in Supporting Table 1; strains and plasmids constructed are listed in Supporting Table 2. To construct a strain producing recombinant CspBA (1-548 aa, expression construct used for antibody production), primers #457 and #459 were used to amplify the sequence encoding amino acids 1- 548 of CspBA from *C. difficile* 630 genomic DNA. The amplified DNA was digested with NcoI and XhoI, ligated to pET28a, and transformed into DH5α. The resulting pET28a-*cspB*(1-548 aa) plasmid was transformed into BL21(DE3). To mutate the catalytic Ser of CspBA, PCR SOE was used to introduce the S461A catalytic Ser point mutation into pET28a-*cspB*(1-548 aa). Primers #457 and 465 were used to amplify a 5’ fragment, and primers #459 and #464 were used to amplify a 3’ fragment. The resulting fragments were used as the templates for a second PCR reaction using the flanking primers, #457 and #459, to amplify *cspB*(1-548 aa) carrying the S461A mutation. A similar strategy was used to mutate the P3-P1 residues of CspBA to produce plasmid pET28a-*cspB*(1-548)-QTQ/AAA, with the exception that the SOE primers were #502 and #503.

To construct a *cd1433* expression construct for antibody production, primers #522 and #523 were used to amplify the *cd1433* gene lacking the stop codon. The resulting PCR product was digested with NcoI and XhoI, ligated to pET28a, and transformed into DH5α. The resulting pET28a-*cd1433* plasmid was transformed into BL21(DE3). To construct a *sleC* expression construct for antibody production, primers #461 and #463 were used to amplify the *sleC* gene lacking the stop codon. The resulting PCR product was digested with NdeI and XhoI, ligated to pET22b, and transformed into DH5α. The resulting pET22b-*sleC* plasmid was transformed into BL21(DE3). To construct a *cspC* expression construct for antibody production, primers #524 and #525 were used to amplify the *cspC* gene lacking the stop codon. The resulting PCR product was digested with NdeI and XhoI, ligated to pET22b, and transformed into DH5α. The resulting pET22b-*cspC* plasmid was transformed into BL21(DE3).

To construct a strain producing recombinant CspB *perfringens*, primers #512 and #513 were used to amplify *cspB* lacking the stop codon from from *C. perfringens* ATCC 13124 genomic DNA (a kind gift of Jimmy Ballard). The PCR product was digested with NdeI and XhoI, ligated to pET22b, and transformed into DH5α. The resulting pET22b-*cspB perfringens* plasmid was transformed into BL21(DE3). To construct a strain expressing the mature form of CspB *perfringens* carrying a C-terminal His6-tag, primers #670 and #671 were used to amplify *cspB* missing the N-terminal 96 aa and carrying a C-terminal His6­-tag. The PCR product was digested with NdeI and XhoI and ligated to pRSFduet1 digested with the same enzymes. The resulting pRSFduet1-*cspB* ∆96 (*C. perfringens*) plasmid was transformed into BL21(DE3). This plasmid was also used to clone a series of transcomplementation constructs. Specifically, primers #668 and #701 were used to amplify the prodomain region of CspB *perfringens* (1-96 aa); primers #668 and #771 were used to amplify aa 1-92 of the CspB *perfringens* prodomain region; primers #668 and #772 were used to amplify aa 1-93 of the CspB *perfringens* prodomain region; primers #703 and #704 were used to amplify the prodomain region of CspC *perfringens*; and primers #457 and #702 were used to amplify the prodomain region of CspBA *difficile* (1-66 aa). A stop codon was added to the 3’ primer of all these prodomain constructs. The resulting PCR products were digested with NcoI and SalI and ligated to pRSFduet1-*cspB* ∆96 plasmid that had been digested with the same enzymes. The resulting plasmid constructs were transformed into BL21(DE3).

To construct a strain expressing full-length CspB *perfringens* carrying a C-terminal His6-tag, primers #512 and #671 were used to amplify *cspB* carrying a C-terminal His6­-tag. The PCR product was digested with NdeI and XhoI and ligated to pRSFduet1 digested with the same enzymes. The resulting pRSFduet1-*cspB* (*C. perfringens*) plasmid was transformed into BL21(DE3). This plasmid was also used to introduce a series of point mutations using site-directed mutagenesis. Primer pair #516 and 517 was used to introduce the catalytic S494A mutation; primer pair #868 and 869 were used to introduce the S96R prodomain mutation; primer pair #709 and 710 was used to introduce the K91D prodomain mutation; primer pair #818 and 819 and primer pair #765 and 766 were used to introduce the R231Q and R231E salt bridge mutations, respectively; primer pairs #820 and 821 and #952 and 953 were used to introduce the E35R and E35Q salt bridge mutations, respectively; and primer pair #822 and 823 was used to introduce the E59A salt bridge mutation. To construct the E35R-R231E salt bridge swap construct, plasmid pRSFduet1-*cspB* R231E was used as the template in a site-directed mutagenesis PCR reaction using primer pair #820 and 821. The resulting plasmids were transformed into BL21(DE3).

PCR SOE was used to construct the YTS_AAA cleavage site mutation construct by using primer pair #512 and 741 to amplify the 5’ fragment and primer pair #740 and 671 to amplify the 3’ fragment. The resulting PCR products were purified and used in a second PCR reaction containing flanking primer pair #512 and 671. The PCR SOE product was digested with NdeI and XhoI and ligated to pRSFduet1 digested with the same enzymes to produce the pRSFduet1-*cspB* YTS/AAA plasmid. A similar strategy was used to delete the P3-P1 residues of CspB *perfringens* to produce plasmid pRSFduet1-*cspB* ∆YTS, with the exception that the SOE primers were #774 and #773. To delete the jellyroll domain of CspB *perfringens*, the same PCR SOE strategy was used except that the SOE primers were #718 and #717. The resulting plasmids were transformed into BL21(DE3).

To construct an expression construct producing the isolated jellyroll domain, primer pair #753 and 754 were used to amplify the region corresponding to aa 293-424 of *cspB* *perfringens*. The resulting PCR product was digested with NdeI and XhoI and ligated into pET22b digested with the same enzymes to produce plasmid pET22b-*cspB* jelly (293-424). This construct was transformed into BL21(DE3).

**Protein Sequencing**. The N-termini of CspB variants were mapped using Edman sequencing and performed by the Protein and Nucleic Acid Facility at Stanford University. Briefly, 10 µg of each CspB variant was resolved by SDS-PAGE and transferred to a PVDF membrane. The membrane was stained with Ponceau S (0.1% w/v, 5% acetic acid), and CspB was excised from the membrane and sequenced using Edman degradation.

**Protein Purification**. For purification of His6-tagged proteins, overnight cultures of the appropriate BL21(DE3) strain were diluted 1:500 in 2L 2YT (5 g NaCl, 10 g yeast extract, 15 g tryptone/L) media and grown shaking (225 rpm) at 37ºC. When an OD600 of 0.6-0.9 was reached, IPTG was added to 250 µM, and cultures were grown for 12-16 hr at 19ºC. Cultures were pelleted, resuspended in 25 mL lysis buffer [500 mM NaCl, 50 mM Tris-HCl, pH 7.5, 15 mM imidazole, 10% v/v glycerol] and flash frozen in liquid nitrogen. Lysates were thawed, then lysed by sonication and cleared by centrifugation at 15,000 x g for 30 minutes. His6-tagged proteins were affinity purified by incubating the lysates in batch with 1.0 mL Ni-NTA Agarose beads (Qiagen) with shaking for 3 hr at 4ºC. The binding reaction was pelleted at 1,500 x g, the supernatant was set aside, and the pelleted Ni-NTA agarose beads were washed 3 x with lysis buffer. His6-tagged proteins were eluted from the beads by the addition of 350 µL high imidazole elution buffer [500 mM NaCl, 50 mM Tris-HCl, pH 7.5, 175 mM imidazole, 10% v/v glycerol]. The elution was repeated four times; the eluate was pooled, buffer exchanged in gel filtration buffer [200 mM NaCl, 10 mM Tris pH 7.5, 5% v/v glycerol), and concentrated to 750 µL. The concentrated prep was pelleted at 13,000 x g for 10 min at 4˚C prior to loading on a Superdex 200 10/30 column (GE Healthcare).

For crystallization studies, C-terminally His6-tagged CspB *perfringens* was affinity purified as described above then gel purified using a HiPrep S200 16/60 Sephacryl column (GE Healthcare); the gel filtration buffer was 150 mM NaCl, 10 mM Tris pH 7.5. The purified protein was concentrated to 10 mg/mL. A single peak was observed by gel filtration, with the elution volume indicating that CspB likely exists as a monomer based on a calibration curve determined using gel filtration standards (data not shown). Consistent with this observation, the elution volume was independent of protein concentration.Protein purity was analyzed by SDS-PAGE followed by Coomassie staining (GelCode Blue, Pierce).

To prepare seleno-methionine (SeMet) substituted CspB for crystallization studies, an overnight culture of B384(DE3) harboring pET22b-CspB *perfringens* was grown in 60 mL minimal media [7.5 mM (NH4)2SO4, 30 mM KH2PO4, 60 mM K2HPO4, 800 mg/L 19 amino acids excluding methionine, 5% w/v glucose, 1 mM MgSO4, 4 mg/L thiamine, 4 mg/L D-biotin] supplemented with 5% LB media. The overnight culture was diluted 1:100 in 6 L minimal media supplemented with 100 mg/L SeMet (Sigma) and grown for 6 hr until an OD600 of 0.6 was reached. Cultures were induced with 250 µM IPTG and grown at 30ºC for an additional 3 hr. SeMet-substituted CspB was purified using a similar protocol to native CspB with the exception that SeMet-substituted CspB was first purified on a 5 mL HiTrap Q anion-exchange column before gel filtration. Affinity-tagged SeMet-substituted CspB was buffer-exchanged into 10 mM Tris-HCl pH 7.5 (buffer A) using an Amicon Ultra-10 (Millipore) before loading it onto the HiTrapQ column; it was eluted over 20 column volumes using 0.75 M NaCl, 10 mM Tris-HCl pH 7.5 as buffer B. Fractions containing SeMet-labeled CspB *perfringens* were pooled, buffer-exchanged into the 150 mM NaCl, 10 mM Tris-HCl pH 7.5 gel filtration buffer, and purified by gel filtration.

**Table S1.** Interactions between prodomain and mature CspB and jellyroll domain and CspB subtilase domain as determined by PDBe PISA .

Prodomain Interaction Interface with Mature Subtilase

| **Hydrogen Bonds** | | | |  |  | |  | |  | |  | |
| --- | --- | --- | --- | --- | --- | --- | --- | --- | --- | --- | --- | --- |
| **##** | **Prodomain** | | | | **Dist. [Å]** | **Subtilase** | | | | | |  |
| 1 | 10 | ASP | OD2 | | 2.76 | 362 | | TYR | | OH | |  |
| 2 | 61 | LEU | O | | 2.77 | 269 | | TYR | | OH | |  |
| 3 | 88 | GLU | OE1 | | 2.73 | 265 | | LEU | | N | |  |
| 4 | 88 | GLU | OE2 | | 3.24 | 266 | | LEU | | N | |  |
| 5 | 91 | LYS | O | | 2.84 | 226 | | SER | | N | |  |
| 6 | 91 | LYS | N | | 2.88 | 226 | | SER | | OG | |  |
| 7 | 91 | LYS | NZ | | 2.86 | 257 | | ASP | | O | |  |
| 8 | 92 | ILE | O | | 2.9 | 255 | | THR | | OG1 | |  |
| 9 | 93 | LEU | O | | 2.87 | 224 | | ALA | | N | |  |
| 10 | 93 | LEU | N | | 2.74 | 224 | | ALA | | O | |  |
| 11 | 94 | TYR | O | | 2.97 | 254 | | SER | | N | |  |
| 12 | 94 | TYR | N | | 2.88 | 254 | | SER | | O | |  |
| 13 | 95 | THR | OG1 | | 3.47 | 222 | | ARG | | NE | |  |
| 14 | 95 | THR | O | | 2.82 | 222 | | ARG | | NH2 | |  |
| 15 | 95 | THR | N | | 3.01 | 222 | | ARG | | O | |  |
| 16 | 95 | THR | OG1 | | 3.7 | 222 | | ARG | | O | |  |
| 17 | 96 | SER | O | | 3.24 | 183 | | HIS | | NE2 | |  |
| 18 | 96 | SER | OG | | 2.61 | 494 | | SER | | OG | |  |
| 19 | 96 | SER | OXT | | 2.97 | 494 | | SER | | N | |  |
| 20 | 96 | SER | OXT | | 2.82 | 287 | | ASN | | ND2 | |  |
| 21 | 96 | SER | OXT | | 3.38 | 493 | | THR | | N | |  |
| 22 | 96 | SER | N | | 3.35 | 252 | | SER | | O | |  |
| 23 | 96 | SER | OG | | 3.45 | 252 | | SER | | O | |  |

**Salt Bridges**

| **##** | **Prodomain** | | | **Dist. [Å]** | **Subtilase** | | |
| --- | --- | --- | --- | --- | --- | --- | --- |
| 1 | 35 | GLU | OE1 | 3.51 | 231 | ARG | NH2 |
| 2 | 35 | GLU | OE1 | 2.96 | 231 | ARG | NH1 |
| 3 | 35 | GLU | OE2 | 2.97 | 231 | ARG | NH2 |
| 4 | 35 | GLU | OE2 | 3.57 | 231 | ARG | NH1 |
| 5 | 59 | GLU | OE1 | 2.83 | 231 | ARG | NH2 |
| 6 | 59 | GLU | OE1 | 2.89 | 231 | ARG | NE |
| 7 | 59 | GLU | OE2 | 3.18 | 231 | ARG | NE |
| 8 | 91 | LYS | NZ | 3.89 | 257 | ASP | OD2 |

The distance between two atoms is shown for each bond; residue number, residue 3-letter code, and atom name specify each atom involved.

***Jellyroll Domain*** Interaction Interface with Mature Subtilase

| **Hydrogen Bonds** | | | |  |  |  |  |  |
| --- | --- | --- | --- | --- | --- | --- | --- | --- |
| **##** | **Jellyroll** | | | | **Dist. [Å]** | **Subtilase** | | |
| 1 | 291 | SER | | N | 3.26 | 288 | GLU | O |
| 2 | 291 | SER | | N | 3.49 | 290 | ASN | OD1 |
| 3 | 291 | SER | | OG | 2.92 | 288 | GLU | OE2 |
| 4 | 291 | SER | | OG | 3.21 | 287 | ASN | O |
| 5 | 293 | | HIS | ND1 | 2.76 | 288 | GLU | OE1 |
| 6 | 294 | HIS | | ND1 | 3.18 | 425 | SER | O |
| 7 | 294 | HIS | | O | 2.82 | 425 | SER | N |
| 8 | 319 | ASP | | OD2 | 3.28 | 258 | GLY | N |
| 9 | 362 | TYR | | OH | 2.76 | 10 | ASP | OD2 |
| 10 | 370 | ASP | | OD2 | 3.29 | 261 | ASN | ND2 |
| 11 | 372 | GLN | | OE1 | 3.55 | 261 | ASN | ND2 |
| 12 | 406 | ASP | | OD2 | 3.03 | 428 | ASN | ND2 |
| 13 | 408 | TRP | | NE1 | 2.89 | 428 | ASN | O |
| 14 | 409 | LEU | | O | 3.46 | 256 | ASN | ND2 |
| 15 | 420 | ARG | | NH2 | 2.84 | 290 | ASN | O |

| **Salt Bridges** | |  |  |  |  |  |  |
| --- | --- | --- | --- | --- | --- | --- | --- |
| **##** | **Jellyroll** | | | **Dist. [Å]** | **Subtilase** | | |
| 1 | 406 | ASP | OD1 | 2.74 | 462 | ARG | NH1 |
| 2 | 406 | ASP | OD1 | 3.46 | 462 | ARG | NH2 |
| 3 | 406 | ASP | OD2 | 3.77 | 462 | ARG | NH1 |
| 4 | 406 | ASP | OD2 | 3.01 | 462 | ARG | NH2 |

***Jellyroll Domain Interaction Interface with Prodomain***

**Hydrogen Bonds**

| **##** | **Jellyroll** | | | **Dist. [Å]** | **Prodomain** | | |
| --- | --- | --- | --- | --- | --- | --- | --- |
| 1 | 362 | TYR | O | 2.76 | 10 | ASP | OD2 |

The distance between two atoms is shown for each bond; residue number, residue 3-letter code, and atom name specify each atom involved.

**Table S2. Strains and plasmids used in this study.**

| **Strain**  **#** | ***C. difficile* strain** | **Relevant genotype or features** | **Source/**  **reference** |
| --- | --- | --- | --- |
| 11 | JIR8094 | Erm-sensitive derivative of 630 | C. Ellermeier |
| 13 | 630 | Clinical isolate 630 | T. Lawley |
| 30 | *cspBAC–* | JIR8094 *cspBAC*::*ermB* | This study |
| 47 | *sleC–* | JIR8094 *sleC*::*ermB* | This study |
| 84 | JIR8094/pMTL84151 | JIR8094/pMTL84151 | This study |
| 88 | *cspBAC–*/pMTL84151 | JIR8094 *cspBAC*::*ermB/* pMTL84151 | This study |
| 111 | JIR8094/pMTL83151 | JIR8094/ pMTL83151 | This study |
| 117 | *cspBAC*–/pMTL83151 | JIR8094 *cspBAC*::*ermB/* pMTL83151 | This study |
| 113 | *cspBAC*–/pMTL83151-*cspBAC* | JIR8094 *cspBAC*::*ermB*/ pMTL83151-*cspBAC* | This study |
| 193 | *cspBAC*–/pMTL83151-*cspBAC* S461A | JIR8094*cspBAC*::*ermB*/pMTL83151-*cspBAC* S461A | This study |
| 197 | *cspBAC*–/pMTL83151-*cspBAC* ∆jelly | JIR8094 *cspBAC*::*ermB*/pMTL83151-*cspBAC* ∆jelly | This study |
| 228 | *cspBAC*–/pMTL84151-*cspBAC* Q66/S461A | JIR8094 *cspBAC*::*ermB*/pMTL84151-*cspBAC* Q66/S461A | This study |
| 234 | *cspBAC*–/pMTL84151-*cspBAC* Q66 | JIR8094 *cspBAC*::*ermB*/pMTL84151-*cspBAC* Q66 | This study |

| ***E. coli* strains with *C. difficile constructs*** | |  |
| --- | --- | --- |
| **Strain (#)** | **Relevant genotype or features** | **Source/**  **reference** |
| DH5a | F– Φ80*lacZ*ΔM15 Δ(*lacZYA–argF*) U169 *recA1 endA1 hsdR17* (rK–, mK+) *phoA supE44* λ– *thi-1 gyrA96 relA1* | D. Cameron |
| BL21(DE3) | F– *ompT hsdSB*(rB–, mB–) *gal dcm* (DE3) | Novagen |
| HB101 | F- *mcrB mrr hsdS20*(rB– mB–) *recA13 leuB6 ara-13 proA2 lavYI galK2 xyl-6 mtl-1 rpsL20* | C. Ellermeier |
| 531 | pK424 in HB101 | C. Ellermeier |
| 7 | pET22b in DH5a | D. Higgins |
| 269 | pET28a in DH5a | M. Bogyo |
| 548 | pRSFDuet1 in DH5a | Novagen |
| 556 | pJS107 in DH5a | J. Sorg |
| 686 | pMTL83151 in HB101/pK424 | This study |
| 655 | pMTL83151 in DH5a | This study |
| 455 | pET28a-*cspB*(548aa) in BL21(DE3) | This study |
| 471 | pET28a-*cspB*(548aa)-S461A in BL21(DE3) | This study |
| 493 | pET28a-*cspB*(548aa)-QTQ/AAA in BL21(DE3) | This study |
| 514 | pET28a-*cd1433* in BL21(DE3) | This study |
| 516 | pET22b-*cspC* (CD2246) in BL21(DE3) | This study |
| 533 | pET22b-*sleC* in BL21(DE3) | This study |
| 604 | pJS107 *cspBA* 81 in HB101/pK424 | This study |
| 646 | pJS107-*sleC* 128 in HB101/pK424 | This study |
| 667 | pMTL83151-*cspBAC* in HB101/pK424 | This study |
| 799 | pMTL83151-*cspBAC* S461A in HB101/pK424 | This study |
| 800 | pMTL83151-*cspBAC* ∆jelly in HB101/pK424 | This study |
| 891 | pMTL84151-*cspBAC* Q66 in HB101/pK424 | This study |
| 892 | pMTL84151-*cspBAC* Q66/S461A in HB101/pK424 | This study |

| ***E. coli* strains with *C. perfringens* constructs** | |  |
| --- | --- | --- |
| **Strain #** | **Relevant genotype or features** | **Source or reference** |
| 506 | pET22b-*cspB* in BL21(DE3) | This study |
| 673 | pRSFduet1-*cspB*∆96 in DH5a | This study |
| 674 | pRSFduet1-*cspB*∆96 in BL21(DE3) | This study |
| 711 | pRSFduet1-*cspB* in BL21(DE3) | This study |
| 712 | pRSFduet1-c*spB*(1-96)/*cspB*∆96 in BL21(DE3) | This study |
| 713 | pRSFduet1-c*spBA difficile* (1-66)/*cspB*∆96 in BL21(DE3) | This study |
| 714 | pRSFduet1-*cspC perfringens* (1-78)/*cspB*∆96 in BL21(DE3) | This study |
| 742 | pRSFduet1-*cspB* ∆jelly in BL21(DE3) | This study |
| 744 | pRSFduet1-*cspB* ∆jelly/S461A in BL21(DE3) | This study |
| 745 | pRSFduet1-*cspB* K91D in BL21(DE3) | This study |
| 747 | pRSFduet1-*cspB* S494A in BL21(DE3) | This study |
| 753 | pRSFduet1-*cspB* YTS/AAA in BL21(DE3) | This study |
| 786 | pET22b-*cspB* jelly (293-424 aa) in BL21(DE3) | This study |
| 788 | pRSFduet1-c*spB*(1-92)/*cspB*∆96 in BL21(DE3) | This study |
| 789 | pRSFduet1-c*spB*(1-93)/*cspB*∆96 in BL21(DE3) | This study |
| 791 | pRSFduet1-*cspB* R231E in BL21(DE3) | This study |
| 792 | pRSFduet1-*cspB* ∆YTS in BL21(DE3) | This study |
| 793 | pRSFduet1-*cspB* R231Q in BL21(DE3) | This study |
| 820 | pRSFduet1-*cspB* E35R in BL21(DE3) | This study |
| 821 | pRSFduet1-*cspB* E59A in BL21(DE3) | This study |
| 822 | pRSFduet1-*cspB* E35R*-*R231E in BL21(DE3) | This study |
| 823 | pRSFduet1-*cspB* S96R in BL21(DE3) | This study |

| **Plasmids** | **Relevant features** | **Source or reference** |
| --- | --- | --- |
| pET22b | *bla* | Novagen |
| pET28a | *kan* | Novagen |
| pRSFduet1 | *kan* | Novagen |
| pK424 | Tra Mob+; *bla, tet* | C. Ellermeier |
| pJS107 | *C. difficile* Targetron construct based on pJIR750ai (group II intron *ermB*::RAM, *ltrA*); *catP* | J. Sorg |
| pCE245 | *C. difficile* Targetron construct based on pJIR750ai (group II intron *ermB*::RAM, *ltrA*); *catP* | C. Ellermeier |
| pMTL83151 | pCB102, Tra+; *catP* | N. Minton |
| pMTL84151 | pCD6, Tra+; *catP* | N. Minton |

**Table S3. Primers used in this study.**

| **#** | **Name** | **Sequence** |
| --- | --- | --- |
| 457 | 5' NcoI *cspBA* start | ATACCATGGCTATTATAATAAATTATGAATTAATTGTA |
| 459 | 3' XhoI *cspBA* 548 aa | GCACTCGAGTGACTTATTAATACTTCTATATCC |
| 461 | 5' NdeI *sleC* | AGCCATATGCAAGATGGTTTCTTAACAGTAAGC |
| 463 | 3' XhoI *sleC* no stop corr | GCACTCGAGAATTAAAGGATTTAAAGAAGCTATTCTAGT |
| 464 | 5' S461A SOE | AGTGGAGCATTAACTGGAACT**G**CCATGGCTACACCTCATGTTACA |
| 465 | 3' S461A Roes | TGTAACATGAGGTGTAGCCATGG**C**AGTTCCAGTTAATGCTCCACT |
| 502 | 5' QTQ/AAA *cspBA* SOE | GAATTTATAGAAAAGCCTTTTATATTA**GCGGCTGCG**GATGTGCAAAGTTTTTCA |
| 503 | 3' QTQ/AAA *cspBA* Roes | TTGAAAAACTTTGCACATC**CGCAGCCGC**TAATATAAAAGGCTTTTCTATAAATTC |
| 512 | 5' NdeI *cspB* perf ATCC 13124 | AGCCATATGGAAAATAAAGCTAAGGTTGGC |
| 513 | 3' XhoI *cspB* perf ATCC 13124 | AGCCTCGAGTCTCCTATTAATTAATAATTCCAT |
| 516 | 5' S494A *cspB* perf SOE | GGATTTGATACTAAAAGTGGTACA**G**CAATGGCTGCGCCACAA |
| 517 | 3' S494A *cspB* perf Roes | TTGTGGCGCAGCCATTG**C**TGTACCACTTTTAGTATCAAATCC |
| 522 | 5' NcoI *cd1433* start | AGCCCATGGCAGTGATTTACATGCCAAATTTGCCA |
| 523 | 3' XhoI *cd1433* no stop | AGCCTCGAGGAATTGCCCATAAATACCTTC |
| 524 | 5’ NdeI *cspC* | AGCCATATGGAAAAATCTTATTGTATAATTTATCAAGGT |
| 525 | 3’ XhoI cspC no stop | AGCCTCGAGGAATTGCCCATAAATACCTTC |
| 532 | 3' Universal EBS | CGAAATTAGAAACTTGCGTTCAGTAAAC |
| 533 | 5' EBS2 *cspBA* 81 | TGAACGCAAGTTTCTAATTTCGGTTGCAATCCGATAGAGGAAAGTGTCT |
| 534 | 3' EBS1d *cspBA* 81 | CAGATTGTACAAATGTGGTGATAACAGATAAGTCTATGATGATAACTTACCTTTCTTTGT |
| 535 | 5' IBS1 *cspBA* 81 | AAAAAAGCTTATAATTATCCTTAATTGCCTATGATGTGCGCCCAGATAGGGTG |
| 536 | 5' CDEP692 | GTAAATTCAGATTCTCGGC |
| 537 | 5' CDEP1070a | AAACGTATAAATTAGGAGGG |
| 538 | 3' CDEP1138 | TTCCGCTGGCAGCTTAAGCA |
| 641 | 5' IBS1 *sleC* 128a | AAAAAAGCTTATAATTATCCTTACATTACTTCTTAGTGCGCCCAGATAGGGTG |
| 642 | 3' EBS1d *sleC* 128a | CAGATTGTACAAATGTGGTGATAACAGATAAGTCTTCTTAGGTAACTTACCTTTCTTTGT |
| 643 | 5' EBS2 *sleC* 128a | TGAACGCAAGTTTCTAATTTCGGTTTAATGTCGATAGAGGAAAGTGTCT |
| 665 | 3' XhoI *cspC* cd2246 + TAA | ACAAGCTCGAGCTATAGAGTATTTGCTATCTGTTG |
| 668 | 5' NcoI *cspB* perf ATG | AAAGTCCATGGAAAATAAAGCTAAGGTTGGC |
| 670 | 5' NdeI *cspB* perf ∆96 | AAATGCATATGGCTTATGATAGTAATAGAGCATCATGC |
| 671 | 3' XhoI 6His *cspB* perf | AATACTCGAGTTAGTGGTGGTGGTGGTGGTGTCTCCTATTAATTAATAATTCCATTGTTC |
| 691 | 5' NotI *cspBA* cd2247 upstream | AGAATGCGGCCGCTTCAATTAATTATTGGTATCAAACTCAAAG |
| 701 | 3' SalI *cspB* perf pro TAA | AAATGGTCGACTTATGATGTATATAAAATTTTGGGTAGCTC |
| 702 | 3' SalI *cspBA* diff pro TAA | AAATGTCGACTTACTGAGTCTGTAATATAAAAGGCTTTTC |
| 703 | 5' NcoI *cspC* perf | TTTACCATGGAAACTGTATCTAATAAAGCG |
| 704 | 3' SalI *cspC* perf pro TAA | AAATGTCGACTTAACTTAGAGTATATATTCCTCCGAG |
| 709 | 5' cspB *perf* K91D SOE | CTTCAATATATAGAGCTACCC**G**A**T**ATTTTATATACATCAGCTTATGATAG |
| 710 | 3' cspB *perf* K91D Roes | CTATCATAAGCTGATGTATATAAAAT**A**T**C**GGGTAGCTCTATATATTGAAG |
| 717 | 5' *cspB* perf ∆jelly SOE | GGGAATGAAGGTAATAGTGCT|TCTGTTTATAATACCTTAGGAATCCCT |
| 718 | 3' *cspB* perf ∆jelly Roes | AGGGATTCCTAAGGTATTATAAACAGA|AGCACTATTACCTTCATTCCC |
| 740 | 5' cspB *perf* YTS/AAA SOE | CAATATATAGAGCTACCCAAAATTTTA**GCGGCCGCA**GCTTATGATAGTAATAGAGCATC |
| 741 | 3' cspB *perf* YTS/AAA Roes | GATGCTCTATTACTATCATAAGC**TGCGGCCGC**TAAAATTTTGGGTAGCTCTATATATTG |
| 745 | 5' *cspBA* DIFF ∆jelly SOE | GCTGGAAATAATGCAGATAAAGGA|ACTCAAGAGCTTACTGTAACAGCT |
| 746 | 3' *cspBA* DIFF ∆jelly Roes | AGCTGTTACAGTAAGCTCTTGAGT|TCCTTTATCTGCATTATTTCCAGC |
| 753 | 5' NdeI *cspB* perf jellyroll | AAATCATATGGCTCATCATGTAGGGGGCAAG |
| 754 | 3' XhoI *cspB* perf jellyroll | AAGACTCGAGTGGTTGTAAAAATCTTGTTCTTTCATTTAATCC |
| 765 | 5' R231E *cspB* perf SOE | GCCTTAAGTACACAGCTTATG**GAA**GGTTTAAAATTTTTAATGGATAAAAGTAATG |
| 766 | 3' R231E *cspB* perf Roes | CATTACTTTTATCCATTAAAAATTTTAAACC**TTC**CATAAGCTGTGTACTTAAGGC |
| 771 | 3' SalI *cspB* perf pro 92aa TAA | AATAGTCGACTTAAATTTTGGGTAGCTCTATATATTGAAG |
| 772 | 3' SalI *cspB* perf pro 93aa TAA | AATAGTCGACTTATAAAATTTTGGGTAGCTCTATATATTGAAG |
| 773 | 5' *cspB* perf ∆YTS SOE | CTTCAATATATAGAGCTACCCAAAATTTTA|GCTTATGATAGTAATAGAGCATC |
| 774 | 3' *cspB* perf ∆YTS Roes | GATGCTCTATTACTATCATAAGC|TAAAATTTTGGGTAGCTCTATATATTGAAG |
| 818 | 5' R231Q *cspB* perf SOE | GCCTTAAGTACACAGCTTATG**CAA**GGTTTAAAATTTTTAATG |
| 819 | 3' R231Q cspB perf Roes | CATTAAAAATTTTAAACC**TTG**CATAAGCTGTGTACTTAAGGC |
| 820 | 5' E35R *cspB* perf SOE | TCCCCTAATAATGGAGAGATA**CG**GTTAGTTGTTTTATATGGAGATAATTTTTTAAG |
| 821 | 3' E35R *cspB* perf Roes | CTTAAAAAATTATCTCCATATAAAACAACTAAC**CG**TATCTCTCCATTATTAGGGGA |
| 822 | 5' E59A cspB perf SOE | GATGTCATAGGTGCTAAAGTTG**C**AGATTTAGGATATGGATTTGGAATAC |
| 823 | 3' E59A *cspB* perf Roes | GTATTCCAAATCCATATCCTAAATCT**G**CAACTTTAGCACCTATGACATC |
| 864 | 3' *cspBA* KpnI 2775-2751 | TGCTAGGCTACTGGTACCTAAAGAC |
| 868 | 5' S96R *cspB* perf SOE | GAGCTACCCAAAATTTTATATACA**CG**AGCTTATGATAGTAATAGAGCATCATGC |
| 869 | 3' S96R *cspB* perf Roes | GCATGATGCTCTATTACTATCATAAGCT**CG**TGTATATAAAATTTTGGGTAGCTC |
| 950 | 5' cspBA Q66+TAG RBS ATG | GACTCAGTAGGAGGGATTTATGGATGTGCAAAGTTTTTCAAGTACAGG |
| 951 | 3' cspBA Q66+TAG RBS Roes | AAATCCCTCCTACTGAGTCTGTAATATAAAAGGCTTTTCTATAAATTCAATTTC |
| 952 | 5' E35Q *cspB* perf SOE | TCCCCTAATAATGGAGAGATA**CA**GTTAGTTGTTTTATATGGAGATAATTTTTTAAG |
| 953 | 3' E35Q *cspB* perf Roes | CTTAAAAAATTATCTCCATATAAAACAACTAAC**TG**TATCTCTCCATTATTAGGGGA |
|  |  |  |

Restriction enzyme sequences are underlined; point mutations are in bold italics; deletion sites are indicated by |.

**Table S4. Data collection and refinement statistics.**

|  | **SeMet CspB** |
| --- | --- |
| **Data collection** |  |
| Space group | P212121 |
| Cell dimensions |  |
| *a*, *b*, *c* (Å) | 73.87, 138.17, 140.08 |
|    γ () | 90, 90, 90 |
| Wavelength (Å) |  |
| Resolution (Å) | 50-1.6 (1.618-1.6)* |
| *R*merge (%) | 7.8 (45.1)* |
| *I* / *I* | 18.43 (2.05)* |
| Completeness (%) | 94.66 (70.0)* |
| Redundancy | 6.6 (2.8)* |
|  |  |
| **SAD Phasing Statistics+** |  |
| Selenium sites (ShelX/D) | 12 |
| PATFOM (/D) | 24.47 |
| Overall CC (ShelX/E) (%) | 46.27 |
| Pseudo-Free CC (/E) (%) | 81.61 |
| Contrast/Connectivity (/E) | 0.803/0.957 |
| Final Map CC (/E) | 0.936 (0.936)* |
|  |  |
| **Refinement** |  |
| Resolution (Å) | 43-1.6 |
| No. reflections | 1,332,184 total  202,733 unique |
| *R*work / *R*free | 0.15/0.18 |
| No. atoms | 9766 |
| Protein | 8772 |
| Ligand/ion | 37 |
| Water | 957 |
| *B*-factors (Å2) | 28.6 |
| Wilson B | 19.1 |
| Protein | 27.5 |
| Ligand/ion | 33.5 |
| Water | 38.2 |
| R.m.s. deviations |  |
| Bond lengths (Å) | 0.0065 |
| Bond angles () | 0.844 |

*Values in parentheses are for highest-resolution shell.

+Statistics for phasing as defined by ShelX/C/D/E program authors4,12.

**Supporting References**

1. Heap JT, Pennington OJ, Cartman ST, Carter GP, Minton NP (2007) The ClosTron: a universal gene knock-out system for the genus *Clostridium*. J Microbiol Methods 70: 452-464.

2. Ho TD, Ellermeier CD (2011) PrsW is required for colonization, resistance to antimicrobial peptides, and expression of extracytoplasmic function sigma factors in *Clostridium difficile*. Infect Immun 79: 3229-3238.

3. O'Connor JR, Lyras D, Farrow KA, Adams V, Powell DR, et al. (2006) Construction and analysis of chromosomal Clostridium difficile mutants. Mol Microbiol 61: 1335-1351.

4. Heap J, Pennington O, Cartman S, Minton N (2009) A modular system for *Clostridium* shuttle plasmids. J Microbiol Methods 78: 79-85.

5. Horton RM, Hunt HD, Ho SN, Pullen JK, Pease LR (1989) Engineering Hybrid Genes without the Use of Restriction Enzymes - Gene-Splicing by Overlap Extension. Gene 77: 61-68.

6. Burns DA, Heap JT, Minton NP (2010) SleC is essential for germination of *Clostridium difficile* spores in nutrient-rich medium supplemented with the bile salt taurocholate. J Bacteriol 192: 657-664.

7. Krissinel E, Henrick K (2007) Inference of macromolecular assemblies from crystalline state. J Mol Biol 372: 774-797.

8. Sebaihia M, Wren BW, Mullany P, Fairweather NF, Minton N, et al. (2006) The multidrug-resistant human pathogen *Clostridium difficile* has a highly mobile, mosaic genome. Nat Genet 38: 779-786.

9. Sheldrick GM (2010) Experimental phasing with SHELXC/D/E: combining chain tracing with density modification. Acta Crystallogr D Biol Crystallogr 66: 479-485.

10. Adams PD, Afonine PV, Bunkoczi G, Chen VB, Davis IW, et al. (2010) PHENIX: a comprehensive Python-based system for macromolecular structure solution. Acta Crystallogr D Biol Crystallogr 66: 213-221.
